# Germline mutations and somatic inactivation of *TRIM28* in Wilms tumour

**DOI:** 10.1371/journal.pgen.1007399

**Published:** 2018-06-18

**Authors:** Benjamin J. Halliday, Ryuji Fukuzawa, David M. Markie, Richard G. Grundy, Jackie L. Ludgate, Michael A. Black, Jane E. Skeen, Robert J. Weeks, Daniel R. Catchpoole, Aedan G. K. Roberts, Anthony E. Reeve, Ian M. Morison

**Affiliations:** 1 Department of Pathology, Dunedin School of Medicine, University of Otago, Dunedin, New Zealand; 2 Department of Pathology, International University of Health and Welfare, School of Medicine, Narita, Japan; 3 Children’s Brain Tumour Research Centre, University of Nottingham, Nottingham, United Kingdom; 4 Cancer Genetics Laboratory, Department of Biochemistry, University of Otago, Dunedin, New Zealand; 5 Starship Children's Hospital, Auckland, New Zealand; 6 Tumour Bank, Children’s Cancer Research Unit, The Children’s Hospital at Westmead, Westmead, NSW, Australia; University College London, UNITED KINGDOM

## Abstract

Wilms tumour is a childhood tumour that arises as a consequence of somatic and rare germline mutations, the characterisation of which has refined our understanding of nephrogenesis and carcinogenesis. Here we report that germline loss of function mutations in *TRIM28* predispose children to Wilms tumour. Loss of function of this transcriptional co-repressor, which has a role in nephrogenesis, has not previously been associated with cancer. Inactivation of *TRIM28*, either germline or somatic, occurred through inactivating mutations, loss of heterozygosity or epigenetic silencing. *TRIM28*-mutated tumours had a monomorphic epithelial histology that is uncommon for Wilms tumour. Critically, these tumours were negative for TRIM28 immunohistochemical staining whereas the epithelial component in normal tissue and other Wilms tumours stained positively. These data, together with a characteristic gene expression profile, suggest that inactivation of *TRIM28* provides the molecular basis for defining a previously described subtype of Wilms tumour, that has early age of onset and excellent prognosis.

## Author summary

The germline and somatic molecular events associated with Wilms tumour, a childhood kidney cancer, have been progressively defined over the past three decades. Among the uncharacterised tumours are a group of tumours that have monomorphic epithelial histology, familial association, distinctively clustered gene-expression patterns, early age of diagnosis, and excellent prognosis. Here, we describe germline mutations and loss of function of *TRIM28* in familial Wilms tumours, along with somatic loss of function in a non-familial Wilms tumour. All *TRIM28*-mutant tumours showed the rare monomorphic epithelial histology, suggesting that loss of *TRIM28* expression could be a useful marker to define a group of tumours with excellent prognosis. Future studies could lead to identification and reassurance of families that carry *TRIM28* mutations, and to the use of reduced intensity of treatment for children who develop *TRIM28*-null tumours.

## Introduction

The study of Wilms tumour, a rare childhood kidney tumour [1], has facilitated the discovery of mechanisms of organogenesis and the neoplastic transformation of embryonic tissue. First, the discovery of inactivating mutations and deletions of *WT1* in Wilms tumours [2] led to the revelation of its key roles in development of numerous embryonic tissues [3, 4]. Similarly, activating mutations of *CTNNB1* in Wilms tumours highlighted the importance of WNT pathway activation in renal development and in multiple tumour types [5]. In addition, altered expression of the imprinted *IGF2* locus demonstrated the occurrence of genomic imprinting in humans, as well as the consequences of its disruption during embryogenesis [6, 7]. Mutations in microRNA processors *DGCR8*, *DROSHA*, and *DICER1* have underscored the importance of this pathway in developmental tumours [8–11], whereas mutations in *SIX1* and *SIX2* reflect their critical role in renal development [9, 10, 12]. Characterisation of other recently reported recurrent somatic mutations [9, 10, 13] will further clarify the mechanisms of nephrogenesis and neoplasia.

Familial and syndromic Wilms tumours have demonstrated the susceptibility of the developing kidney to germline variants of *WT1* in children with genitourinary abnormalities [14], *BRCA2* and *PALB2* in Fanconi anaemia patients [15, 16], *GPC3* in Simpson-Golabi-Behmel syndrome patients [17], *DIS3L2* in Perlman syndrome [18], *DICER1* in DICER1-related disease [19], *BUB1B* and *TRIP13* in mosaic variegated aneuploidy (MVA) syndrome [20, 21], and *CTR9* [22], *REST* [23], *PALB2*, and *CHEK2* [13] in non-syndromic Wilms tumour families. In addition, linkage of familial Wilms tumours to 17q12-q21 [24] and 19q13.4 [25] implicate further causative gene variants, although the evidence supporting the 19q13.4 locus was not conclusive [26].

Molecular characterisation of Wilms tumours has assisted in the stratification of tumours into clinically relevant subgroups [27]. For example, children with tumours with diffuse anaplasia, associated with *TP53* mutations [28], are recommended to receive more intense therapy [27]. In addition, losses of chromosomal arms 1p or 16q are associated with poorer outcomes [29] and augmented therapy has been recommended [27]. Conversely, small stage 1 tumours with favourable histology in young children can be treated with less intense regimens [27]. Over-represented in this last group are a cluster of tumours (S1) described by Gadd and colleagues that do not harbour mutations in *WT1*, *CTNNB1* or *AMER1*. These tumours usually show retention of imprinting at *IGF2*, have a distinct gene expression pattern and have highly differentiated monomorphic epithelial histology [30, 31].

Additional characterisation of Wilms tumour subtypes by molecular events and gene expression should enable the refinement of clinically significant risk categories and enhance therapeutic outcomes. Here we report the presence of truncating germline variants, somatic mutation, and epigenetic silencing of *TRIM28*, in familial and non-familial cases of Wilms tumour. Tumours with these alterations have the characteristic histology, gene expression and outcome typical of the previously described S1 subtype [30].

## Results

### *TRIM28* mutations and methylation

We performed whole-exome sequencing on Wilms tumours and matched adjacent kidney from 18 unrelated patients. Following processing of the sequence reads to variant calls, we first assessed the non-neoplastic kidney sequences for rare germline variants using a candidate gene approach. Genes containing variants previously associated with Wilms tumour and genes within regions of familial linkage, 17q12-q21 and 19q13.4, were included in this analysis.

**Fig 1:**
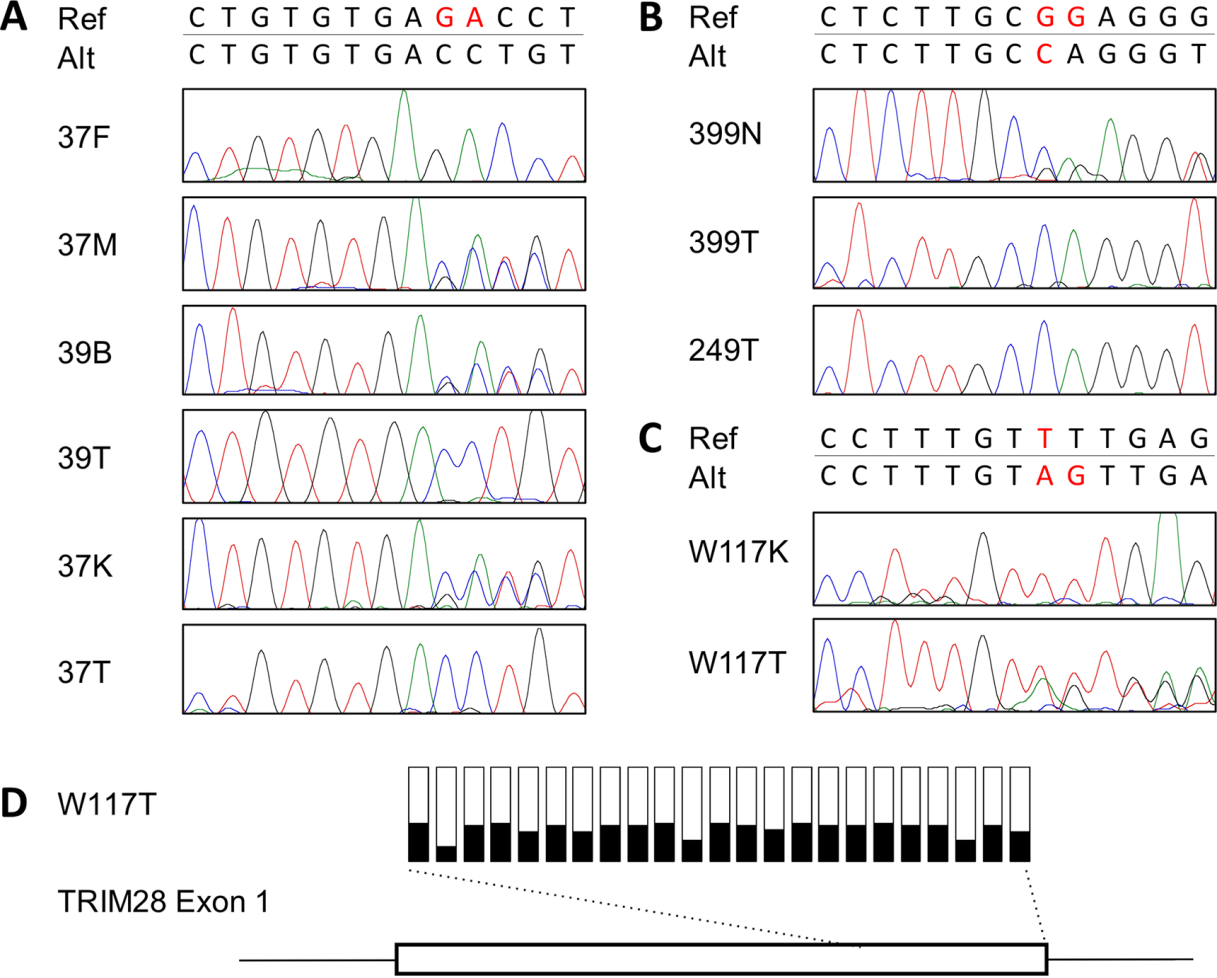
DNA sequence and methylation of *TRIM28*. (**A**) Family 1. 2-bp deletion (c.525_526del) in the blood of case 39 (39B), the kidney of case 37 (37K) and the blood of their mother (37M). The father (37F) was unaffected. The tumours from cases 37 and 39 (37T and 39T) showed loss of heterozygosity. (**B**) Family 2. Germline deletion/insertion (c.1746_1747delinsC) in blood DNA from case 399 (399N) with loss of heterozygosity in tumours 399T and 249T. (**C**) Somatic deletion/insertion mutation (c.1935delinsGA) in W117 tumour (W117T) and reference sequence in the adjacent kidney (W117K). (**D**) The proportion of methylated CpGs in exon 1 of *TRIM28* in W117T as measured by targeted bisulfite PCR. For each CpG site the black portion of the bar shows the proportion of methylated reads.

One case (case 37, diagnosed at 39 months) showed a constitutional frameshift variant of *TRIM28* (NM_005762.2; c.525_526del) in the non-neoplastic kidney sample (Table 1, Fig 1A, S1 Text). *TRIM28*, which encodes a transcriptional co-repressor, is located at 19q13.4 in the proximity of a putative familial Wilms tumour locus [25]. Analysis of the sequence of the associated tumour (37T) revealed loss of heterozygosity with retention of the variant allele (Fig 1A, S1 Fig). Peripheral blood DNA from the patient’s sister, who was diagnosed with bilateral Wilms tumours at 8 months of age (case 39), showed heterozygosity for the same 2-bp deletion. DNA was then extracted from one of the paraffin-embedded tumours of case 39 revealing loss of heterozygosity, with retention of the variant allele (Fig 1A). The same 2-bp deletion was also present in peripheral blood from their asymptomatic mother thereby confirming maternal transmission of the *TRIM28* variant. Notably, the mother had no history of cancer.

**Table 1:** Genetic, epigenetic and clinical features of monomorphic epithelial Wilms tumours.

Case number	*TRIM28* mutations*	Protein*	Sex	Age of onset	Features	Outcome
37	c.525_526del in kidney with LOH in tumour	p.(Glu175Aspfs*29)	M	39 m	Unilateral, stage 1, monomorphic epithelial	Alive at 23 years
39	c.525_526del in blood with LOH in tumour	p.(Glu175Aspfs*29)	F	8 m	Bilateral, monomorphic epithelial	Alive at 20 years
Mother of 37 & 39	c.525_526del in blood	p.(Glu175Aspfs*29)	F	NA	No childhood tumour	Alive
W117	c.1935delinsGA and exon 1 methylation in tumour	p.(Phe645Leufs*30)	M	7 m	Unilateral, stage 1, monomorphic epithelial	Alive at 26 years
249	c.1746_1747delinsC with assumed LOH in tumour	p.(Glu583Argfs*93)	M	8 m	Stage 1, monomorphic epithelial	Alive at 30 years
399	c.1746_1747delinsC with LOH in tumour	p.(Glu583Argfs*93)	F	5 m	Stage 1, monomorphic epithelial	Alive at 29 years
SCTBN 88	No mutation or methylation detected		F	18 m	Stage 1, monomorphic epithelial. PLNR^†^	Unknown

* NM_005762.2

† PLNR, perilobar nephrogenic rest

Loss of function (LoF) variants of *TRIM28* are exceedingly rare. To determine the prevalence of these events in the population, we interrogated the gnomAD database (http://gnomad.broadinstitute.org/) which contains sequence data for more than 140,000 individuals. In total four LoF variants were detected, two of which are described as low confidence variants. In addition, the probability of LoF intolerance (pLI) for *TRIM28* was 1.0 [ExAC database (http://exac.broadinstitute.org/)], where pLI ≥ 0.9 indicates extreme LoF intolerance [32]. Furthermore, *TRIM28* is constrained with respect to missense variation, having a constraint z score of 3.16 (ExAC database) indicating high intolerance to variation [32].

Tumour-kidney pairs were then analysed for acquired somatic pathogenic mutations (S1 Table). Among these 18 pairs, a heterozygous frameshift mutation in exon 13 of *TRIM28* (c.1935delinsGA) was detected in a sporadic tumour (W117) (Table 1, Fig 1C), though a second inactivating mutation or deletion could not be detected from the exome data. On inspection of exon read-depth it was noted that exon 1 was not represented in the aligned exome sequences despite being included in the capture platform. In addition, exon 1 was intractable to standard PCR approaches, presumably because of its high GC content (greater than 80%).

To overcome this issue, W117 tumour DNA was bisulfite-treated to reduce the GC content of the template, and Sanger sequence was produced for both treated DNA strands to determine mutational status. No variants were detected, but extensive methylation across a 480-bp portion of the CpG island that flanks exon 1 (S2 Fig) was discovered. Massively-parallel sequencing of bisulfite-converted DNA was then used to quantify methylation, revealing dense methylation of all CpGs throughout the amplified 220-bp region in 39% of 1043 sequence reads (Fig 1D; S3 Fig). In histologically normal adjacent kidney tissue, exon 1 methylation was also detected in 1.2% of sequence reads whereas the exon 13 mutation was not detected, suggesting low level mosaicism for *TRIM28* hypermethylation (S4 Fig). In contrast, seven other Wilms tumours, including five with similar histology, showed absence of methylation in this region (S2 Fig). In addition, three normal kidney samples showed absence of methylated *TRIM28* alleles.

The observations of a heterozygous frameshift truncating mutation and a heterozygous region of dense exon 1 CpG island methylation suggest that both alleles of *TRIM28* have been inactivated, although it cannot be formally excluded that the mutation and CpG island methylation affect the same allele.

Remarkably, the tumours from case 37, case 39 (sister of case 37) and case W117 shared the same rare monomorphic epithelial histological pattern that occurs in approximately 5% of Wilms tumours [30]. We, therefore, sought other tumours to determine whether loss of function of TRIM28 was a shared feature of monomorphic epithelial tumours.

A literature search to find other similar tumours identified a family involving an affected mother and two children with monomorphic epithelial Wilms tumours (Table 1, cases 249 and 399) [33]. Targeted Sanger sequencing of all *TRIM28* exons was achieved using tumour DNA from both children and the blood DNA of case 399. A frameshift variant in exon 13 (c.1746_1747delinsC) was detected in the blood DNA of case 399 and in the tumours from the children. Both tumours showed loss of the non-variant allele (Fig 1B). DNA from the mother’s tumour or normal tissue was not available for sequencing.

We then identified a sporadic tumour with monomorphic epithelial histology among 92 Wilms tumours in the Sydney Children’s Tumour Bank Network (SCTBN). Targeted Sanger sequencing of all *TRIM28* exons of this tumour (SCTBN 88) did not identify any mutations and exon 1 was unmethylated.

### Loss of TRIM28 expression

**Fig 2:**
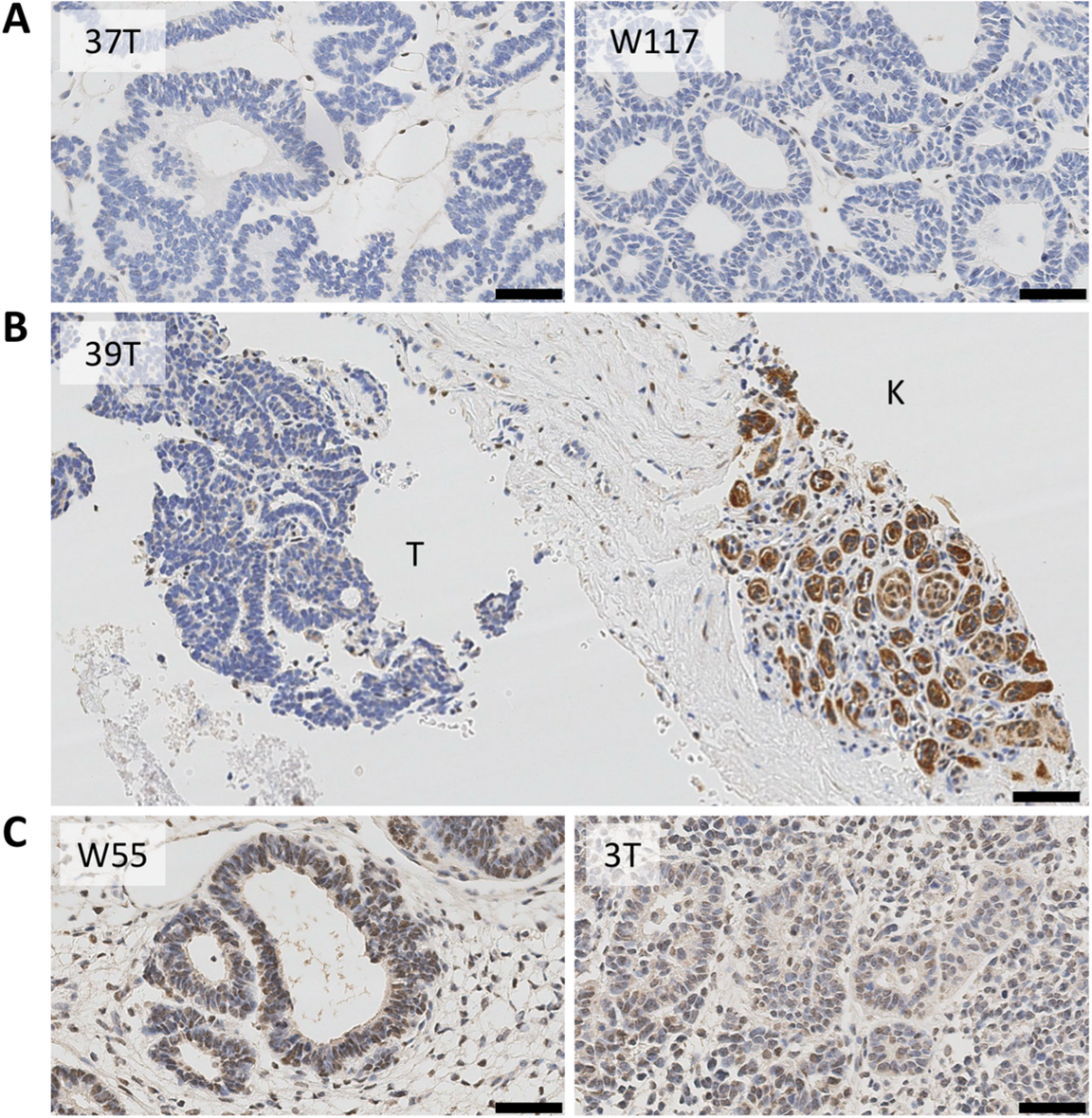
TRIM28 immunohistochemistry. (**A**) Monomorphic epithelial Wilms tumours showing absence of TRIM28 expression in 37T and W117. (**B**) Absence of TRIM28 protein expression in tumour (T) but not in adjacent kidney (K) in case 39. (**C**) Positive control showing TRIM28 expression in two representative Wilms tumours. Black line = 50 μM.

Immunohistochemistry for TRIM28 protein was done for tumours 37T, 39T and W117 to determine whether mutations in *TRIM28* led to loss of protein expression. All three tumours had a complete absence of TRIM28 protein in neoplastic cells (Fig 2A & 2B), although non-tumour-derived endothelial cells and residual non-neoplastic kidney epithelial structures (K) showed positivity. Nine other Wilms tumours were examined and all showed immunohistochemical expression of TRIM28 in epithelial elements, examples of which are shown in Fig 2C. Tumour SCTBN 88, which also showed monomorphic epithelial histology but no *TRIM28* mutations, exhibited a normal pattern of TRIM28 expression by immunohistochemistry.

### No other mutations detected in tumours with *TRIM28* variants

**Fig 3:**
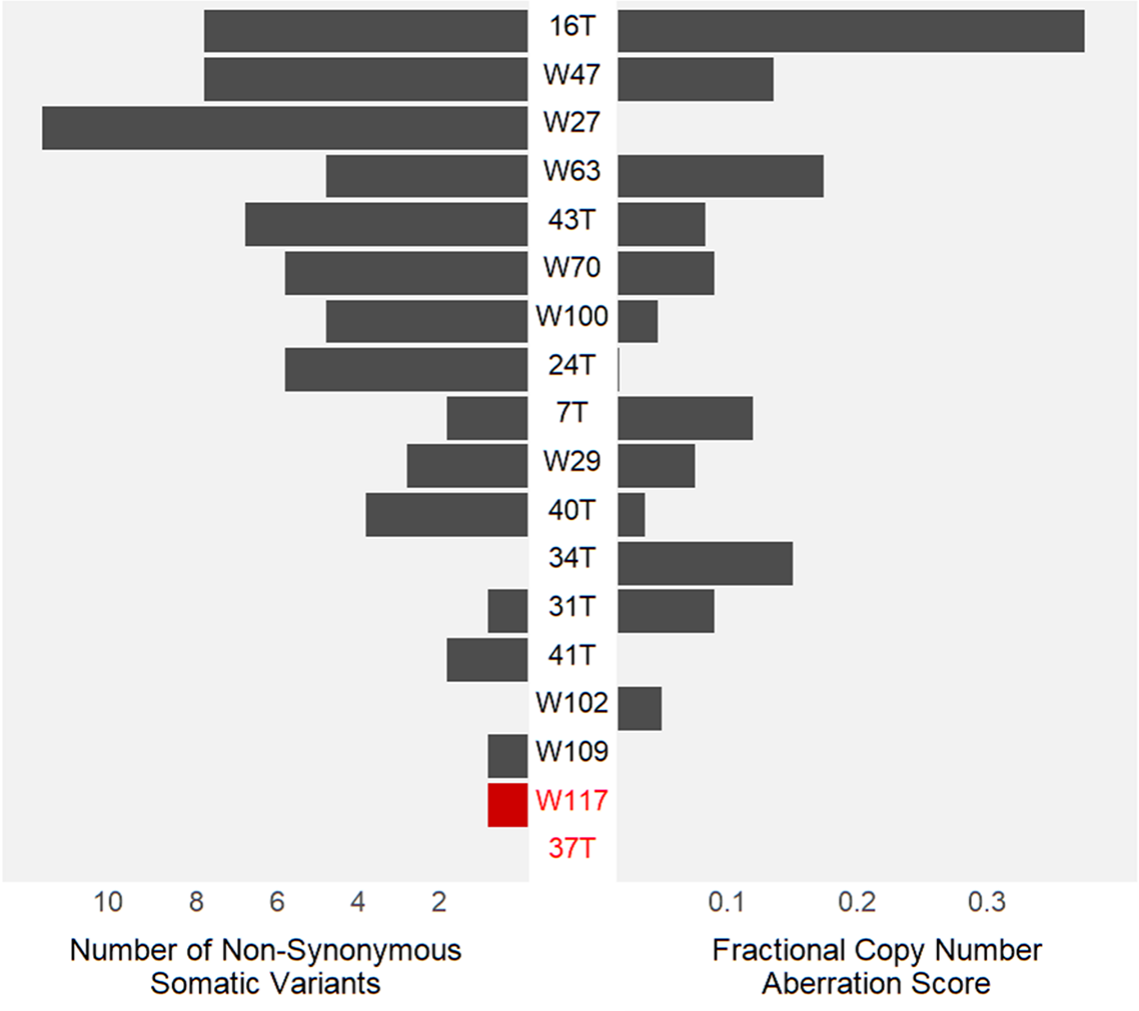
Somatic genetic changes in Wilms tumours. The left side shows the number of somatic non-synonymous and truncating mutations for each tumour detected by MuTect2. The single somatic variant in W117 is the *TRIM28* mutation. The right side shows the fractional length of aberrant copy number segments as determined by ADTEx.

Whole-exome sequencing of 37T and W117 revealed no somatic mutations of other genes known to be mutated in Wilms tumour, including *WT1*, *AMER1*, *CTNNB1*, *DROSHA*, *DGCR8*, *SIX1*, *SIX2* and *REST*. Indeed, no additional missense or non-functional mutations that passed standard filtering criteria were detectable in any other gene in these tumours. By comparison, the 16 sequenced tumours without *TRIM28* variants had a mean and median of 4 (range, 0–12) detected somatic variants (Fig 3).

Exome sequencing data were then used to detect copy number change and loss of heterozygosity in the Wilms tumours (ADTEx, http://adtex.sourceforge.net). Tumour 37T showed copy-neutral loss of heterozygosity at fourteen contiguous SNPs from chr19:59023166 (hg19) to chr19:qter (chr19:59,118,983) without copy number variation, consistent with homozygosity of the inherited variant (S5 Fig). The most distal heterozygosity on 19q was detected at chr19:59,010,819 (rs2278497); therefore, the homozygous region includes the genes *SLC27A5*, *ZBTB45*, *TRIM28*, *MIR6807*, *CHMP2A*, *UBE2M*, *MZF1*, and *MZF1-AS1*.

Apart from loss of heterozygosity within 19q13.43, tumour 37T showed no other chromosomal regions with copy number changes or loss of heterozygosity, above the baseline noise level. The fractional length of aberrant copy number segments was quantified using segmentation data obtained with ADTEx, based on exome sequencing read depth. Tumour W117 also showed no evidence of regional gains or losses or loss of heterozygosity throughout the sequenced genome. In comparison, 12 of 16 other tumours without *TRIM28* variants showed extensive copy number change (Fig 3, S6 Fig). The fractional length of aberrant copy number segments across the genome was 0.0003 in both 37T and W117 compared to a median of 0.06 for all tumours (Fig 3). The remarkable genomic simplicity of tumours 37T and W117 provides strong evidence that loss of *TRIM28* is the sole driver of tumorigenesis in these cases.

### Gene expression is consistent with that of the “S1” subgroup

**Fig 4:**
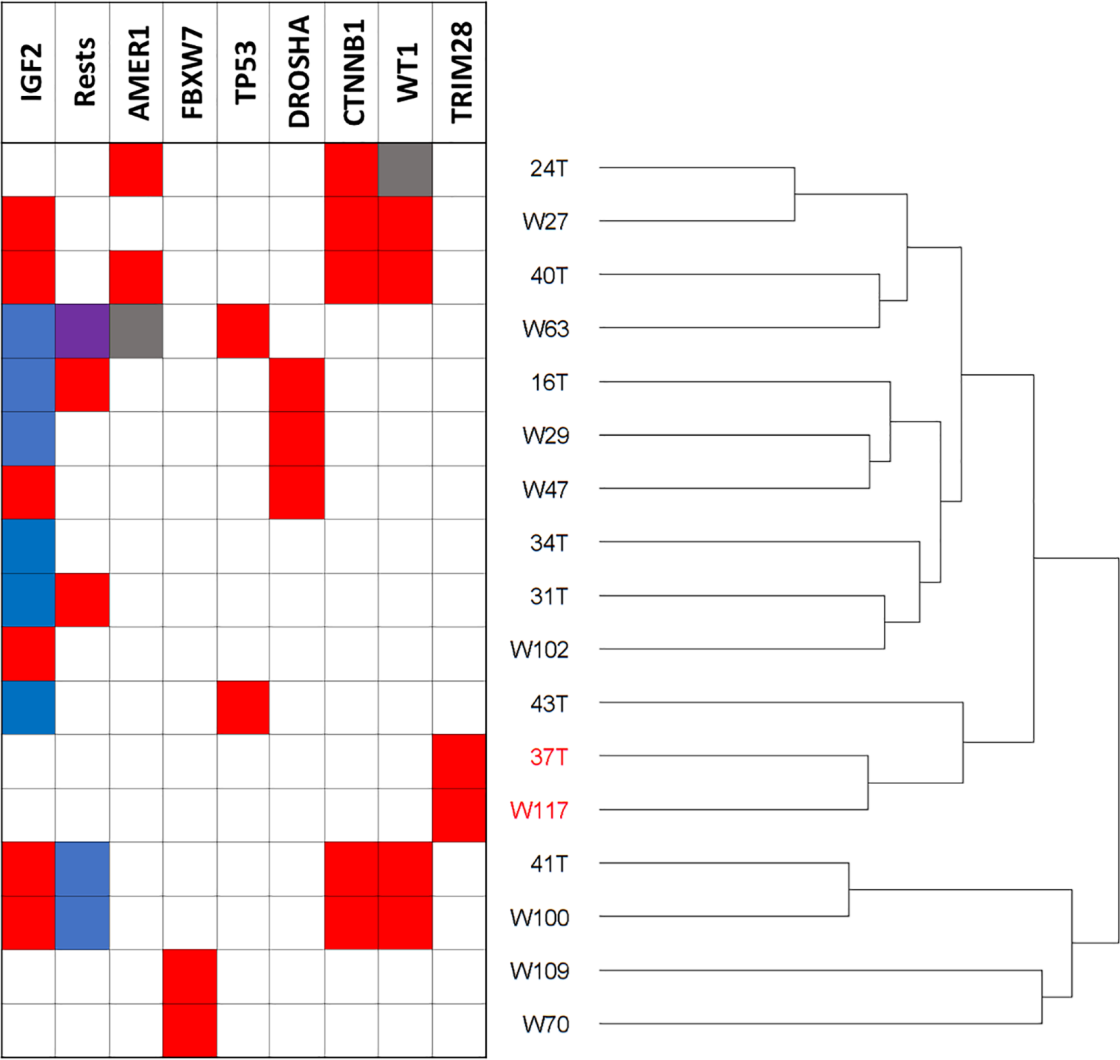
Dendrogram from unsupervised hierarchical clustering of gene expression of 17 Wilms tumours. IGF2, refers to *IGF2* status where blue = loss of imprinting, and red = loss of heterozygosity at *IGF2*. Rests refers to the presence of nephrogenic rests (NR) were blue = intralobar NR, red perilobar NR and purple NR of unknown type. For each gene, red boxes indicate the presence of mutation, whereas the grey box denotes gene deletion.

**Fig 5:**
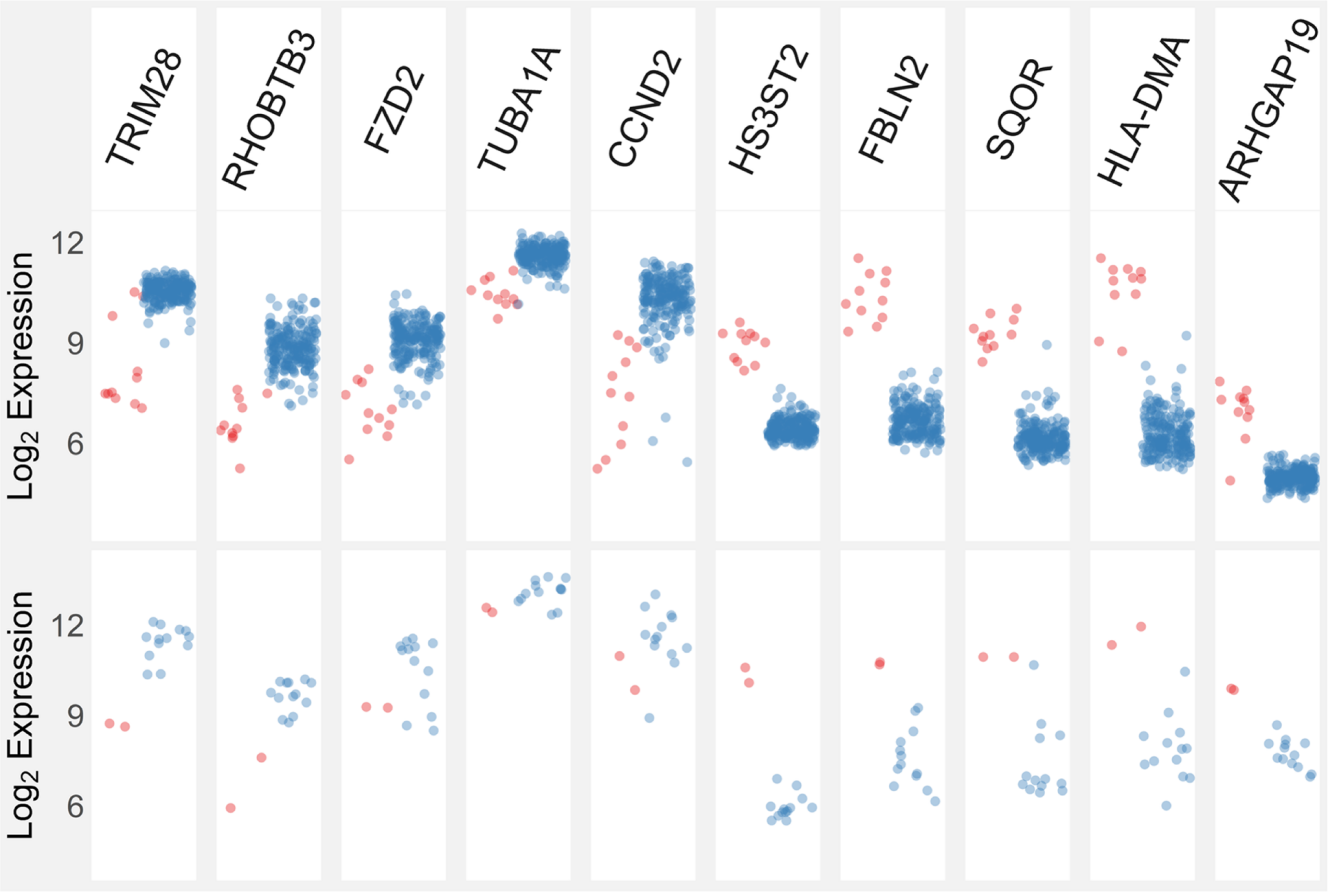
Comparison of gene expression between S1 and other Wilms tumours. The upper panels show the five most down-regulated and five most up-regulated genes in the S1 subgroup (n = 11) compared to S2-S5 tumours (n = 213) in the study of Gadd et al. [30]. The lower panels show expression of these genes in *TRIM28*-mutated tumours and 13 other tumours from this study. Red circles, S1 or *TRIM28*-mutated tumours. Blue circles, favourable histology tumours. Note that two tumours with anaplastic histology, both of which had *TP53* mutations, are not included to maintain comparability with the favourable histology tumours reported by Gadd et al. [30].

The gene expression of 17 of these 18 Wilms tumours had previously been obtained using Affymetrix Human Genome U133 Plus 2.0 Arrays [34]. Unsupervised hierarchical clustering of tumour samples using 25,387 probes showed that the two *TRIM28*-mutated tumours for which RNA was available (37T and W117) clustered together (Fig 4). In agreement with the lack of TRIM28 protein, the expression of *TRIM28* mRNA (probe 200990_at) in 37T and W117 was substantially lower than in the other Wilms tumours, consistent with complete or marked loss of expression (Fig 5).

We then compared the gene expression of 37T and W117 to that previously described for the “S1” subgroup of Wilms tumours that have a distinctive monomorphic epithelial histology [30]. First, we examined the gene expression data (GSE31403, Affymetrix Human Genome U133A Array) from the publication of Gadd and colleagues that described 224 cases of favourable histology Wilms tumour [30]. To facilitate comparison with our tumour cohort, we identified and ranked the probes that showed the greatest difference in gene expression between S1 tumours (n = 11) and the S2-S5 tumours (n = 213). Using the data from Gadd and colleagues, we identified 2476 and 2085 probes that showed higher and lower expression respectively in the S1 tumours compared to the other tumours (analysed using limma software with an adjusted p value cut-off of 0.05, Benjamini and Hochberg adjustment) [30]. Similarly, in the *TRIM28*-mutant tumours (with less statistical power) 80 probes showed significantly higher expression than non-mutant tumours, whereas 19 probes showed lower expression.

Of the probes that showed higher and lower expression in the *TRIM28*-mutant tumours, 51 (64%) and 15 (79%) were included in the differentially expressed probes from the data of Gadd et al. [30]. The expression levels of the five most down-regulated and five most up-regulated genes in the S1 compared to S2-S5 subgroups were examined in the *TRIM28*-mutant and non-mutant tumours (Fig 5). The gene expression pattern is remarkably similar, suggesting that *TRIM28*-mutant tumours 37T and W117 have the gene expression characteristics of the S1 subgroup. Indeed, the probe showing the most significant down regulation in S1 tumours compared to the other tumours was probe 200990_at that targets *TRIM28*. Furthermore, in eight of the 11 S1-subtype tumours the expression level of *TRIM28* was distinctly lower than that in all the 213 non-S1 tumours (Fig 5).

We then determined whether the differentially expressed genes (S1 vs S2-S5) elucidated the processes of tumorigenesis or the tissue composition of the S1 tumours. We selected the 302 genes that showed at least two-fold higher expression in S1 compared to S2-S5 tumours and an adjusted p < 0.01. We similarly selected 126 genes that had lower expression in S1 tumours. Pathway and process enrichment analysis (http://metascape.org/) of the 302 over-expressed and 126 under-expressed genes revealed enrichment of several unrelated biological processes (S2 Table) from which convincing conclusions could not be drawn about the mechanisms of tumorigenesis. Instead, we adopted a targeted approach by examining the expression of genes shown to be involved in the different stages of nephrogenesis as documented by the GenitoUrinary Development Molecular Anatomy Project [35]. Marker genes that are highly expressed at each of several specific stages of kidney development were used to create “metagenes” that represent the expression pattern of a matrix of genes [36]. S1 tumours showed significantly higher metagene scores for marker genes expressed during stage I / stage II nephron development (including renal vesicle, comma-shaped body and s-shaped body development) (S7 Fig). This association was largely driven by *LHX1*, *CDH4*, *BMP2*, *POU3F3*, *CCND1*, and *JAG1*. In contrast, marker genes for stage III and IV nephron development, including renal corpuscle and proximal tubule development were not associated with S1 tumours. Therefore, the monomorphic epithelial elements of the S1 tumours are developmentally equivalent to renal-vesicle-derived structures and not to mature epithelial elements.

### Loss of TRIM28 did not affect genomic imprinting

TRIM28 has numerous roles as a transcriptional co-repressor, including involvement in the establishment of imprinting [37, 38]. Therefore, we examined the allelic expression of *H19* and *IGF2*, genes known to be aberrantly imprinted in some Wilms tumours. *IGF2* had normal monoallelic expression in both tumours (37T and W117). In addition, *H19* was monoallelically expressed indicating retention of normal imprinting in tumour 37T. Together with the observations of retention of normal epigenetic status at *IGF2*/*H19* in the previously published S1 subgroup [30], our observations suggest that the tumorigenic effects of *TRIM28* variants are not mediated through defects in the establishment or maintenance of imprinting at the *IGF2*/*H19* locus.

### Lack of association with *AMER1* mutations

Since it has been reported that TRIM28 interacts with AMER1 (WTX) [39] we postulated that tumours with mutations in *AMER1* might share common features with *TRIM28*–mutated tumours. Neither of the *TRIM28*-mutated tumours (37T and W117) had *AMER1* mutations. Following unsupervised clustering analysis of genome-wide gene expression (Fig 4) the *TRIM28* and *AMER1*-mutated tumours did not cluster together. Furthermore *AMER1*-mutated tumours did not show the characteristic histological features of *TRIM28*-variant tumours in our cohort, nor in that of Gadd and colleagues [30]. Therefore, there is no evidence to suggest that *TRIM28* and *AMER1* variants are functionally equivalent in Wilms tumour, or affect related pathways of tumorigenesis.

## Discussion

Here we report that mutations of *TRIM28*, a gene located in proximity of the candidate familial Wilms tumour locus on 19q13.4, are present in the germline in families with Wilms tumours. Remarkably, all four familial and one sporadic *TRIM28*-inactivated tumours had monomorphic epithelial morphology. Their morphology and gene expression pattern accord with those reported for the “S1” subgroup of tumours that have an early age of onset and for which causative mutations have not yet been identified [13, 30]. Previous genome-wide sequencing studies of Wilms tumours, which have targeted high risk blastemal tumours [10] and relapsed or anaplastic tumours [9, 13], did not reveal any germline *TRIM28* variants in Wilms tumours although a single somatic *TRIM28* splice-site mutation has been detected in a *TP53*-mutated tumour with diffuse anaplastic histology [13].

High levels of *TRIM28* expression occurs in many tumour types [40], but loss of TRIM28 function has not previously been implicated in human cancer. The combination of frameshift mutations and loss of heterozygosity or promoter methylation of the non-variant allele indicates complete loss of TRIM28 function in the tumours, that was confirmed by immunohistochemistry. Critically, TRIM28 appears to be essential for normal nephrogenesis, in that silencing of Trim28 in cultured rat kidney rudiments resulted in branching arrest of the ureteric bud structures [41]. It is plausible that loss of ureteric bud development leads to a failure to inhibit the growth of early epithelial structures from the undifferentiated metanephric mesenchyme. Current models of kidney development suggest, however, that differentiation and growth of the earliest nephron-associated structures rely on inductive signals from the ureteric bud tips, the absence of which is associated with failure of nephrogenesis [42, 43]. TRIM28 is known to contribute to the regulation of a wide range of cellular processes including suppression of retrotransposons, regulation of gene expression through heterochromatisation, mediation of DNA damage response, stimulation of epithelial mesenchymal transition and maintenance of stem cell pluripotency [40], highlighting multiple paths by which inactivation of *TRIM28* might induce Wilms tumorigenesis.

Wilms tumours are reported to have a low mutation burden. For example, Wegert and colleagues detected an average of 6 (0–15) non-synonymous somatic mutations, including missense, stop loss, stop gain, and splicing mutations in 58 blastemal type tumours by exome sequencing [10]. Similarly, Walz and colleagues [9] reported an average of 11 high-quality non-synonymous somatic mutations in favourable histology tumours (range 2–42). Here we report a mean of four (range 0–12) high quality somatic variants per tumour, but unusually the two *TRIM28*-mutant tumours analysed by exome sequencing revealed no additional mutations. Using an exome-sequencing-based analysis, there were no meaningful structural changes in these tumours except, in one case, copy-neutral loss of heterozygosity at 19q13.43 which encompasses *TRIM28*. The absence of other identifiable genomic changes in two *TRIM28*-inactivated tumours suggests that loss of *TRIM28* might be the sole driver of tumorigenesis. As such these Wilms tumours could represent rare examples of the “two-hit” model of Wilms tumorigenesis predicted by Knudson [44].

Interactions of TRIM28 with other known Wilms tumour-associated proteins raise the possibility of functional links to tumorigenesis. For example, TRIM28 has been identified as a binding partner of REST [45], which is known to have germline or somatic mutations in approximately 2% of Wilms tumours [23]; however, reported tumours with *REST* mutations had more varied histology and older ages at diagnosis than our group of *TRIM28*-mutant tumours. TRIM28 has also been reported to co-immunoprecipitate with AMER1, which is mutated or deleted in 20–30% of Wilms tumours [46]. The expression patterns of *AMER1* and *TRIM28* mutant tumours did not, however, cluster together, nor did they show similar histological features, suggesting that these two proteins contribute to different tumorigenic pathways.

The clinical behaviour of all five *TRIM28*-variant tumours supports previous observations that the monomorphic epithelial subtype of Wilms tumour is usually associated with excellent prognosis and presentation with early stage disease [30].

However, not all monomorphic epithelial tumours have these features; those that do not, tend to have presentation at later stages of diseases and at an older age [30]. In our study, one monomorphic epithelial tumour had neither *TRIM28* mutations nor loss of *TRIM28* expression. We hypothesise that loss of *TRIM28* expression or the presence of *TRIM28* mutation, in combination with monomorphic epithelial histology, can be used to identify the good prognosis S1 subtype of tumours. If this hypothesis is supported by future analysis of S1 tumours, it is likely to provide a molecular basis for down-staging treatment in affected children, thereby minimizing adverse effects of chemotherapy.

## Methods

### Ethics statement

Wilms tumours and normal samples were collected and analysed with approval from the Health and Disability Ethics Committees, Ministry of Health, New Zealand (approval number CTY/01/10/141). Informed verbal consent was given to the treating surgeon or oncologist prior to tumour resection.

### Exome sequencing, processing and analysis

Exome libraries were constructed and sequenced by the Kinghorn Centre for Clinical Genomics (Garvan Institute of Medical Research, Sydney) using an Illumina HiSeq 2500 machine, with prior enrichment using the SeqCap EZ Exome v3 (Roche) capture platform. Sequence reads were paired end, with read lengths of 125 bases.

### Processing and analysis of exome sequence data

Processing for alignment and standard variant calling was based on GATK Best Practice Guidelines (https://software.broadinstitute.org/gatk/best-practices/). GATK version 3.5 was used.

### Alignment of reads

Paired-end reads in fastq format, derived from a single individual, were aligned to the reference sequence (GRCh37 assembly) using the Burrows-Wheeler Aligner v0.7.13 [47] with the mem algorithm. Duplicate reads were identified using Picard MarkDuplicates. The data were locally realigned around indels followed by Base Quality Score Recalibration to produce the aligned files in bam format.

### Identifying germline variants from non-tumour samples

A variant call of single nucleotide variants (SNVs) and short insertions/deletions (indels) were generated for each sample using GATK HaplotypeCaller. Joint genotyping was done using GATK GenotypeGVCFs to produce a standard variant calling dataset containing variant information for all samples. This was followed by GATK LeftAlignAndTrimVariants and then Variant Quality Score Recalibration was undertaken independently for SNPs and indels. To facilitate the filtering of germline variants in the non-tumour samples, SnpEff version 4.2 [48] was used to annotate with gene context information [49]. Annotation for population allele frequencies was added using GATK VariantAnnotator, with data from the 1000 Genomes Project [50], and the Exome Aggregation Consortium [32].

### Identifying somatic variants from tumour samples

Somatic SNVs and indels in tumour samples were called using the MuTect2 workflow (https://software.broadinstitute.org/gatk/best-practices/mutect2.php). Non-tumour samples from 28 individuals, whose exome sequences were obtained using the same capture platform, was used to create a panel of normals to exclude recurrent variants. dbSNP v137 was used as a “red” list, and the COSMIC database v54 as a “white” list in the recommended workflow.

### Regions of somatic copy number variation and loss of heterozygosity in tumours

Copy number variants and loss of heterozygosity (LOH) were assessed in tumours using the ADTEx (Aberration Detection in Tumour Exome) package v2.0 [51]. Initially, for each tumour-normal pair, all biallelic variants that were heterozygous in the normal sample, with a Genotype Quality greater than or equal to 14 and read depth between 11 and 1001 in both the normal and the tumour sample, were extracted from the multi-sample standard variant call file described above.

B-allele fractions were calculated and used in conjunction with the aligned bam files for the tumour-normal pair and a bed file for the SeqCap EZ Exome v3 capture regions, as input for the ADTEx package to identify regions of copy number variation (S6 Fig) and loss of heterozygosity in each tumour. To provide a simple quantitative measure of the genomic regions affected by copy number change, the segmentation data produced by ADTEx was used to estimate the fraction of the genome affected. The total length of segments with copy gain or loss, relative to the total length of segments reported for that tumour, was calculated as the fractional copy number aberration score. The R package ‘ggplot2’[52] was used for visualisation of regions of copy number variation and loss of heterozygosity (S5 and S6 Figs).

### *TRIM28* exon 1 bisulfite sequencing

Genomic DNA was bisulfite converted using EZ DNA Methylation kit (Zymo #D5002) and PCR amplified using KAPA HiFi HotStart Uracil + polymerase (KAPA Biosystems KK2802) and primers designed to a 253 bp region of *TRIM28* exon 1 (GRCh37/hg19 chr19:59056298–59056550) followed by a second round of PCR (10 cycles) to add indexed Illumina sequencing adaptors (S3 Table). Products were then sequenced on an Illumina MiSeq sequencer (Reagent kit V2, Nano). The methylation patterns of reads were visualised using BiQ Analyzer.

### Expression analysis

Tumour mRNA expression data, generated using an Affymetrix HG-U133 Plus 2.0 GeneChip Array, were available for 17 of the tumours in this study [34]. Expression data generated by Gadd and colleagues using an Affymetrix HG-U133A GeneChip Array were downloaded from GEO [53] (accession number GSE31403 [30]). Data were normalised using Robust Multi-array Average algorithm implemented in the ‘affy’ R package [54]. Probe sets from both datasets were filtered independently on inter-sample variance, and the 50% most variable probes were retained. Further, probes with known cross-hybridisation issues were excluded [55]. After filtering, 25387 probe sets were retained from this study’s data, while 9863 probe sets remained from Gadd and colleague’s data.

Hierarchical clustering of tumours was performed using Euclidean distance and average linkage. Differential expression between S1 tumours and non-S1 tumours was detected using the R package 'limma', accounting for multiple comparisons through the BH method [56]. To facilitate comparison between the two datasets, only probe sets present in both datasets that mapped to known genes, were used.

For comparison of gene expression of S1-S5 tumours with kidney development marker genes annotated in the GUDMAP database [35], the expression of each marker gene was scaled to a mean of 0 and standard deviation of 1, a metagene value was determined (based on first eigenvector from Singular Value Decomposition of the marker genes for that developmental stage [36]) and the tumour subtypes were compared. The p values shown in S7 Fig are not corrected for multiple comparisons.

### Immunohistochemistry

TRIM28 immunohistochemistry was performed using an anti-KAP1 rabbit polyclonal antibody (Abcam ab10484) at a 1:2000 dilution, following antigen retrieval at pH 9.

## Supporting information

S1 Text Additional clinical details of the Wilms tumour cases. (DOCX)

S1 Table Missense and loss of function somatic variants identified in the Wilms tumours. (XLSX)

S2 Table Top 20 clusters with their representative enriched terms (one per cluster) associated with genes that had higher expression (n = 302) or lower expression (n = 126) in S1 than S2-S5 tumours (http://metascape.org/). (XLSX)

S3 Table PCR primers used in this study. (XLSX)

S1 Fig Pedigree description for cases with *TRIM28* mutations. Known affection status is annotated on each individual. A depiction of allele status is presented for each child for both germline and tumour samples. A red bar represents a frameshifting mutation, while an orange box represents hypermethylation. Square brackets indicate assumed status. * These tumours showed loss of heterozygosity but it is unknown if the LOH is copy neutral or copy-loss in these cases. ** It cannot be formally excluded that the mutation and CpG island hypermethylation affected the same allele.
(TIFF)

S2 Fig Bisulfite sequencing (reverse strand) of a portion of the CpG island flanking *TRIM28* exon 1. This shows equal peak heights for G and A nucleotides corresponding to an equal proportion of C and T at multiple CpG sites, suggestive of hemimethylation of *TRIM28* in Wilms tumour W117T. No evidence of methylation was detected in adjacent kidney tissue (W117K), parental blood (W117M and W117F) and seven other Wilms tumours (two examples, 88T and 86T, are shown). The sequence traces are reverse sequences using primers complementary to the bisulfite-converted lower strand (TRIM28_Exon1_BiSulf_Positive_3 & 4).
(TIFF)

S3 Fig Methylation sequencing of *TRIM28* in tumour W117. Each row shows one of 1043 alleles sequenced by MiSeq (GRCh37/hg19 chr19:59056298–59056550). Each column shows one of 23 CpG sites within exon 1 and intron 1 of *TRIM28*. 39.5% of sequence reads are densely methylated (red), whereas 60% show unmethylation, consistent with allele-specific methylation.
(TIFF)

S4 Fig Methylation and genomic sequencing of *TRIM28* in kidney adjacent to tumour W117. Bisulfite sequencing of DNA extracted from the kidney adjacent to W117 (W117K-a) revealed that 24 of 1757 (1.4%) sequences were densely methylated at *TRIM28* exon 1. An additional independent sample W117K-b of adjacent kidney was then assessed by microscopy of H&E-stained frozen sections and found to be free of histological evidence of Wilms tumour. DNA, extracted from an adjacent microtome section of this independent sample, was similarly bisulfite converted and sequenced. Of 661 sequences, eight (1.2%) were densely methylated. We also measured the proportion of alleles carrying the exon 13 c.1935delinsGA frameshift mutation by using deep sequencing of the mutated exon. In the first sample 2 of 1077 (0.19%) of alleles carried the mutation, whereas in the independent replicate 0 of 1212 did. These results indicate that approximately 2.4% of cells carry a methylated *TRIM28* allele in the absence of the tumour-defining mutation suggesting that methylation within normal kidney was the first *TRIM28*-inactivating event.
(TIFF)

S5 Fig SNP allelic fraction (mirrored) at 19q13.43 showing the telomeric region of loss of heterozygosity in tumour 37T. (TIFF)

S6 Fig Copy number variation of the 18 Wilms tumours compared to their paired normal kidney samples, as determined by ADTex (Aberration Detection in Tumour Exome) package v2.0. (TIFF)

S7 Fig Analysis of expression of marker genes involved in different stages of nephrogenesis, as documented by the GenitoUrinary Development Molecular Anatomy Project, in tumour subgroups from Gadd et al. [30]. (PDF)
