# Supplementary material for: Germline mutations and somatic inactivation of TRIM28 in Wilms tumour
Source: PLoS Genet. 2018 Jun 18;14(6):e1007399. doi: 10.1371/journal.pgen.1007399 (PMC6005459; doi:10.1371/journal.pgen.1007399)
Supplement: S1 Text — (DOCX) [file pgen.1007399.s001.docx]

**S1 Text. Additional clinical details of the Wilms tumour cases**

Also see S1 Figure

**37T**

Male

Age at diagnosis: 39 months.

Stage 1. Monomorphic epithelial histology. No nephrogenic rests.

Treatment: UK W 3 weekly vincristine x 10 and 3 weekly Actinomycin-D at weeks 1,4, 7 and 10 + nephrectomy.

Weight 82.6 kg, height 176 cm, BMI 26.8 (when last seen).

Alive without recurrence at age 23 years.

**39T**

Female

Age at diagnosis: 8 months.

Stage V (local stages 1 or 2). Monomorphic epithelial histology. No nephrogenic rests.

Treatment UK W3 - weekly vincristine x 11 then 3 weekly Vincristine, Actinomycin-D, Doxorubicin (to 270 mg/m2) till week 26 + bilateral nephron sparing surgery.

Weight 58.3 kg, height 156.7 cm, BMI 23.7 (when last seen).

Alive without recurrence at age 20 years.

**W117**

Male

Age at diagnosis: 7 months.

Stage 1. Monomorphic epithelial histology. No nephrogenic rests.

Tumour weight 630g.

Treated on RCH W 82 weekly vincristine x 10 and 3 weekly Actinomycin-D + nephrectomy.

Alive without recurrence at age 26 years.

**249**

Male

Age at diagnosis: 8 months.

Stage 1. Monomorphic epithelial histology.

Weight 70.8 kg, 172.9 cm, BMI 23.7 at age 15 years.

Alive without recurrence at age 30 years.

**399**

Female

Age at diagnosis: 5 months.

Stage 1. Monomorphic epithelial histology. No nephrogenic rests noted.

138 g kidney and tumour largely replaced by a smooth nodular mass measuring 7 cm in maximum diameter.

Weight 66.9 kg, height 171.2 cm, BMI 22.8 at age 22 years.

Alive without recurrence at age 29 years.

None of the children showed syndromic features.
