# Supplementary figures and images for: Germline mutations and somatic inactivation of TRIM28 in Wilms tumour
Source: PLoS Genet. 2018 Jun 18;14(6):e1007399. doi: 10.1371/journal.pgen.1007399 (PMC6005459; doi:10.1371/journal.pgen.1007399)

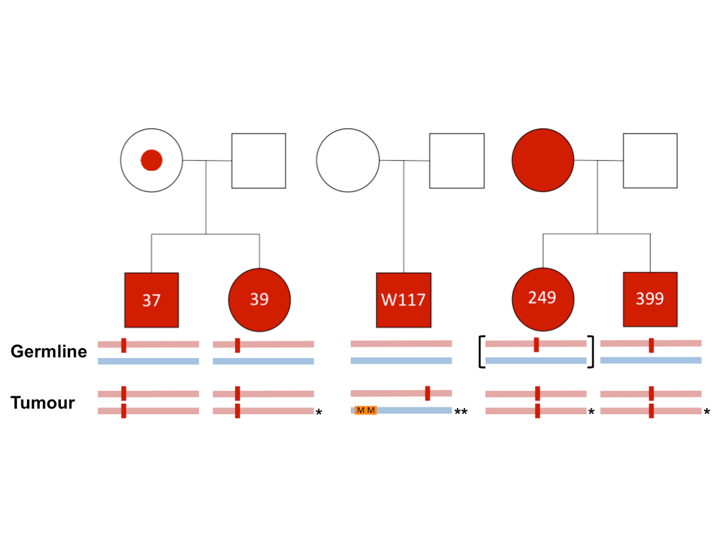

Supplement: S1 Fig — Known affection status is annotated on each individual. A depiction of allele status is presented for each child for both germline and tumour samples. A red bar represents a frameshifting mutation, while an orange box represents hypermethylation. Square brackets indicate assumed status. * These tumours showed loss of heterozygosity but it is unknown if the LOH is copy neutral or copy-loss in these cases. ** It cannot be formally excluded that the mutation and CpG island hypermethylation affected the same allele. (TIFF) [file pgen.1007399.s005.tiff]

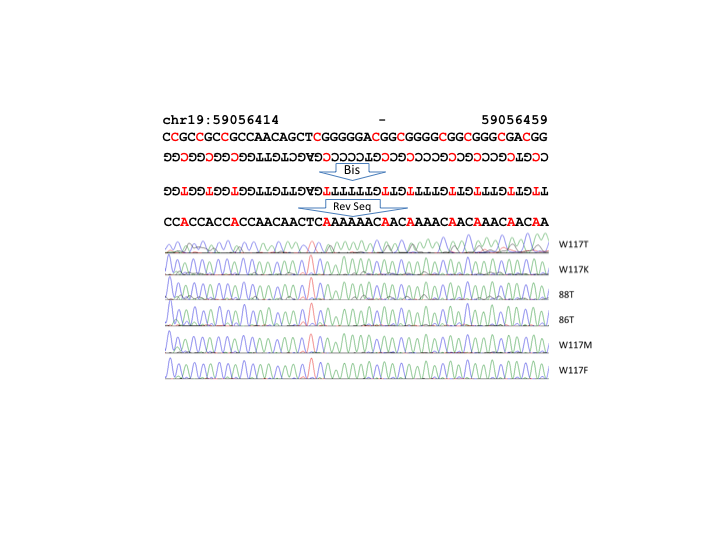

Supplement: S2 Fig — This shows equal peak heights for G and A nucleotides corresponding to an equal proportion of C and T at multiple CpG sites, suggestive of hemimethylation of TRIM28 in Wilms tumour W117T. No evidence of methylation was detected in adjacent kidney tissue (W117K), parental blood (W117M and W117F) and seven other Wilms tumours (two examples, 88T and 86T, are shown). The sequence traces are reverse sequences using primers complementary to the bisulfite-converted lower strand (TRIM28_Exon1_BiSulf_Positive_3 & 4). (TIFF) [file pgen.1007399.s006.tiff]

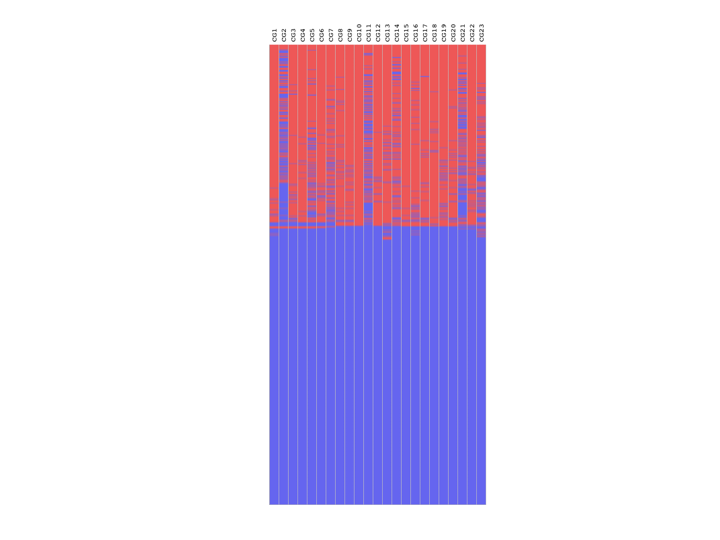

Supplement: S3 Fig — Each row shows one of 1043 alleles sequenced by MiSeq (GRCh37/hg19 chr19:59056298–59056550). Each column shows one of 23 CpG sites within exon 1 and intron 1 of TRIM28. 39.5% of sequence reads are densely methylated (red), whereas 60% show unmethylation, consistent with allele-specific methylation. (TIFF) [file pgen.1007399.s007.tiff]

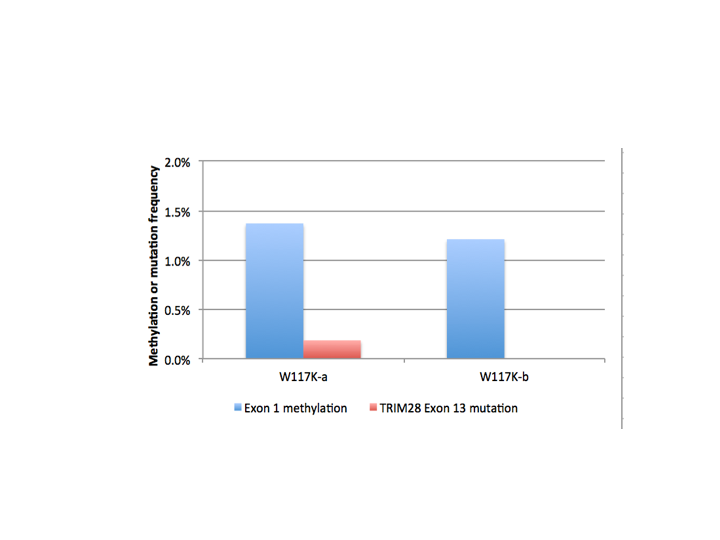

Supplement: S4 Fig — Bisulfite sequencing of DNA extracted from the kidney adjacent to W117 (W117K-a) revealed that 24 of 1757 (1.4%) sequences were densely methylated at TRIM28 exon 1. An additional independent sample W117K-b of adjacent kidney was then assessed by microscopy of H&E-stained frozen sections and found to be free of histological evidence of Wilms tumour. DNA, extracted from an adjacent microtome section of this independent sample, was similarly bisulfite converted and sequenced. Of 661 sequences, eight (1.2%) were densely methylated. We also measured the proportion of alleles carrying the exon 13 c.1935delinsGA frameshift mutation by using deep sequencing of the mutated exon. In the first sample 2 of 1077 (0.19%) of alleles carried the mutation, whereas in the independent replicate 0 of 1212 did. These results indicate that approximately 2.4% of cells carry a methylated TRIM28 allele in the absence of the tumour-defining mutation suggesting that methylation within normal kidney was the first TRIM28-inactivating event. (TIFF) [file pgen.1007399.s008.tiff]

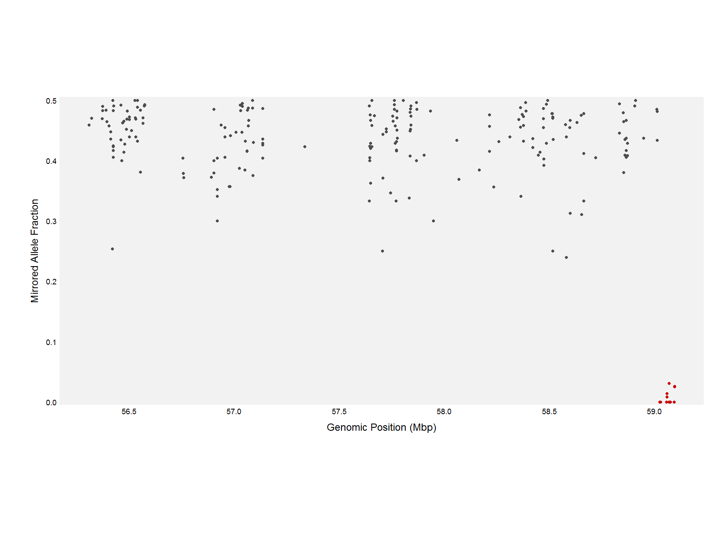

Supplement: S5 Fig — (TIFF) [file pgen.1007399.s009.tiff]

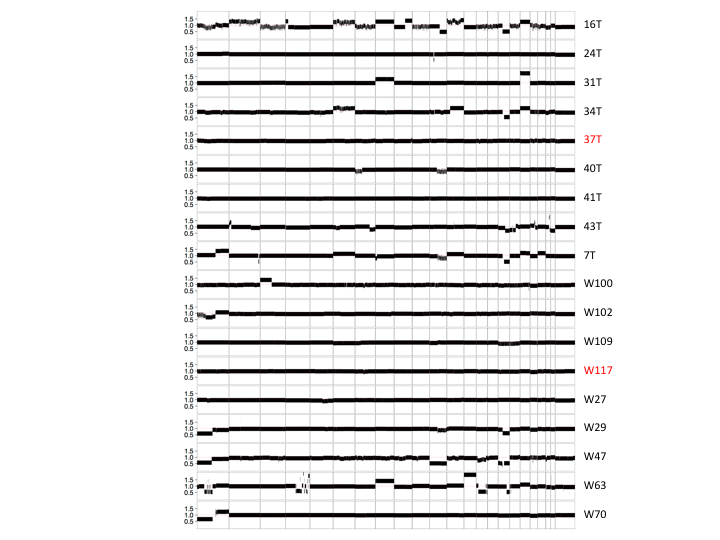

Supplement: S6 Fig — (TIFF) [file pgen.1007399.s010.tiff]
