# Supplementary material for: Germline mutations and somatic inactivation of TRIM28 in Wilms tumour
Source: PLoS Genet. 2018 Jun 18;14(6):e1007399. doi: 10.1371/journal.pgen.1007399 (PMC6005459; doi:10.1371/journal.pgen.1007399)

GUDMAP genes - Gadd et al (2012) data

Summary of analysis

Gene lists

For each of the tissues below, gene lists have been retrieved from the GUDMAP Metanephros page at:

http://www.gudmap.org/Organ_Summaries/index.php (http://www.gudmap.org/Organ_Summaries/index.php)

Tissues:

nephrogenic zone (TS21-TS28)
cap mesenchyme (TS19-TS28)
pretubular aggregate (TS20-TS28)
ureteric tip (TS18-TS28)
stage I / stage II nephron (TS20-TS27)
renal vesicle (TS20-TS27)
comma-shaped body (TS20-TS27)
s-shaped body (TS21-TS27)
stage III / stage IV nephron (TS22-TS28)
renal proximal tubule (TS25-TS28)
loop of Henle (TS25-TS28)
renal distal tubule (TS25-TS28)
renal corpuscle (TS21-TS28)
capillary loop stage nephron (stage III) (TS22-TS28)
maturing nephron (stage IV) (TS22-TS28)
collecting duct (TS21-TS28)
cortical collecting duct (TS22-TS28)
medullary collecting duct (TS22-TS28)
renal interstitium (TS20-TS28)
nephrogenic interstitium (TS20-TS28)
renal cortical interstitium (TS22-TS28)
renal medullary interstitium (TS22-TS28)
pelvis (TS21-TS28)
perihilar interstitium (TS22-TS28)
pelvic smooth muscle (TS23-TS28)
immature loop of Henle (TS22-TS24)
pelvic urothelial lining (TS22-TS28)
early distal tubule (TS21-TS27)
anlage of loop of Henle (TS21-TS27)
small blood vessels (TS23-TS28)

Methods

For each tissue type, “”Anchor" and “Marker” gene information was retrieved, although only the Marker lists are currently used in the analysis below.

Using the Marker list for each tissue, data for these genes was extracted from the Gadd et al (2012) data set (GSE31403: 224 samples).

Per tissue, the following information is presented for the GSE31403 data set:

- an image of the tissue location (taken from GUDMAP)
- a table of Marker genes associated with that tissue. Columns are:
  - mouse gene name,
  - Affymetrix probe IDs associated with human gene (NB - no alias matching performed yet)
  - Affymetrix probe used to represent gene (based on `collapseRows` function from the `WGCNA` package)
- Heatmap of the genes using representative probes:
  - scaled to mean 0, standard devitation 1 per gene (red is high expression, blue low)
  - sorted by increasing metagene value (based on first eigenvector from Singular Value Decomposition). Metagene score is presented above the heatmap as a blue-red bar (ordered).
  - coloured bar above represents tumour subgroups (S1-S5) as defined in the Gadd et al publication (see boxplots for colour legend).
- Boxplots (with overlayed dot plots) of metagene values for each sample across the tumour subgroups.

Results

Based on the outputs presented below, the tissue types with the strongest links to the S1 subgroup are:

- pretubular aggregate (TS20-TS28). Prominent genes: LHX1, CDH4, CCND1
- stage I / stage II nephron (TS20-TS27). Prominent genes: LHX1, CDH4, BMP2, POU3F3, CCND1, JAG1
- renal vesicle (TS20-TS27). Prominent genes: LHX1, CDH4, BMP2, POU3F3, CCND1, JAG1
- comma-shaped body (TS20-TS27). Prominent genes: LHX1, CDH4, BMP2, POU3F3, CCND1, JAG1
- s-shaped body (TS21-TS27). Prominent genes: LHX1, CDH4, BMP2, POU3F3, CCND1, JAG1

In all cases the metagene scores indicated higher levels of expression in the S1 subgroup for many of the tissue-defining genes. It is clear that there is substantial overlap across the gene lists for these tissue types, and that these results are largely being driven by the genes: LHX1, CDH4, BMP2, POU3F3, CCND1, JAG1.

nephrogenic zone (TS21-TS28)

Gene	Probes	Selected
9230110F11Rik		
Ccnd1	208711_s_at, 208712_at	208712_at
Cdh11	207172_s_at, 207173_x_at	207173_x_at
Cdh4	206866_at, 220227_at	206866_at
Csrp1	200621_at	200621_at
Dact1	219179_at	219179_at
Dll1		
Dnm3os		
Eya1	214608_s_at	214608_s_at
Greb1	205862_at, 210562_at, 210855_at	210855_at
Hoxa10	213147_at, 213150_at	213150_at
Kitl		
Lhx1	206230_at	206230_at
Mfap4	212713_at	212713_at
Mll5		
Mmrn1	205612_at	205612_at
Nr2f2	209119_x_at, 209120_at, 209121_x_at, 215073_s_at	209121_x_at
Sox9	202935_s_at, 202936_s_at	202936_s_at
Tgfbi	201506_at	201506_at
Tmem100	219230_at	219230_at
Tpm2	204083_s_at, 212654_at	204083_s_at
Wnt4	208606_s_at	208606_s_at
Wt1	206067_s_at, 216953_s_at	206067_s_at

nephrogenic zone (TS21-TS28)

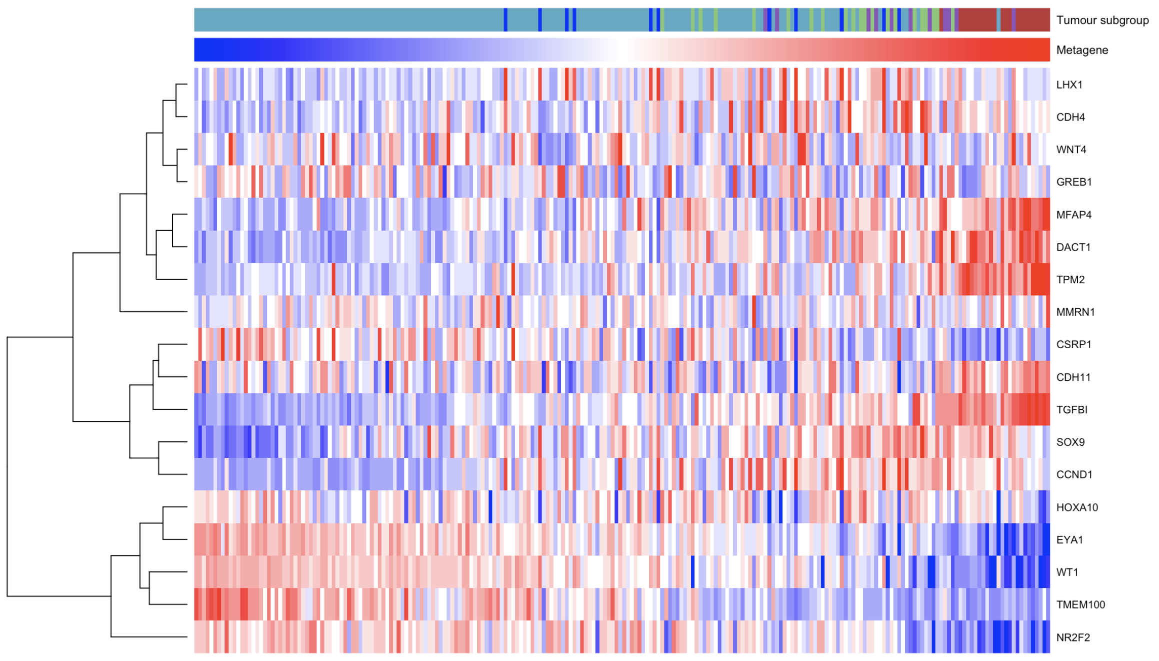

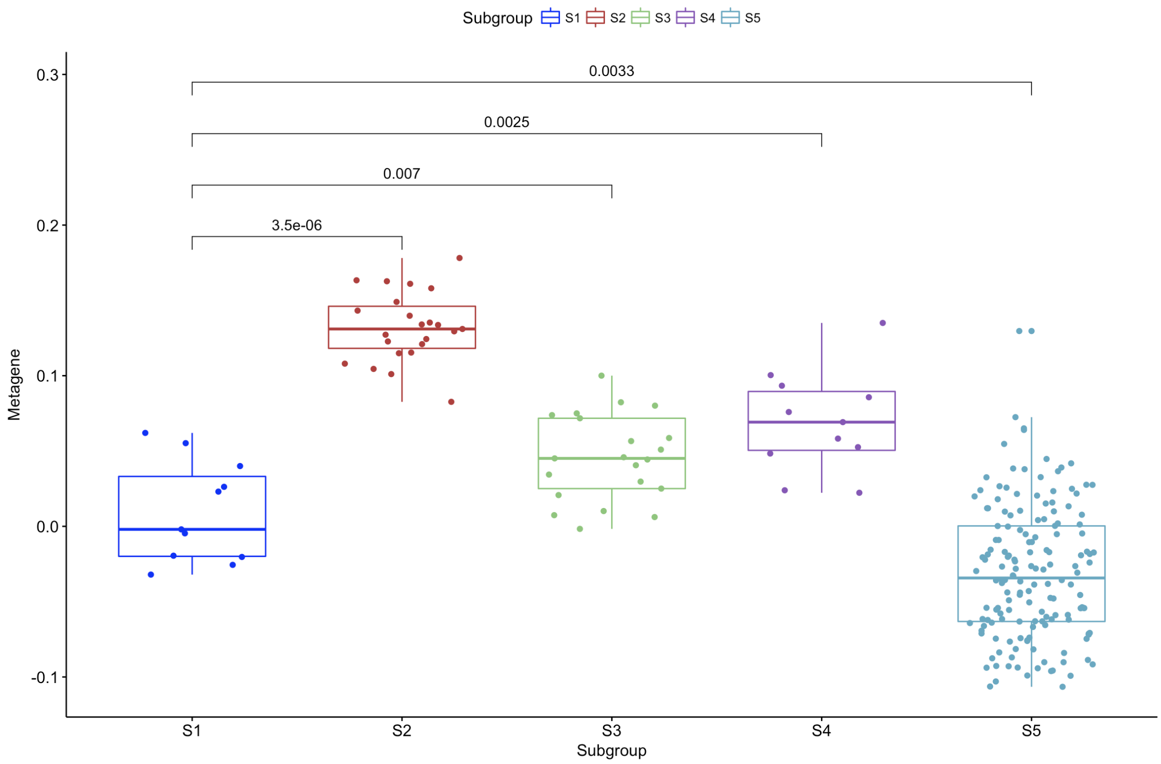

Subgroup S1 S2 S3 S4 S5
0.0033
0.0025
0.007
3.5e-06
0.3
0.2
0.1
0.0
-0.1
Metagene
S1
S2
S3
S4
S5
Subgroup

cap mesenchyme (TS19-TS28)

Gene	Probes	Selected
Cdh11	207172_s_at, 207173_x_at	207173_x_at
Csrp1	200621_at	200621_at
Dact1	219179_at	219179_at
Dnm3os		
Eya1	214608_s_at	214608_s_at
Hoxa10	213147_at, 213150_at	213150_at
Mfap4	212713_at	212713_at
Mll5		
Mmrn1	205612_at	205612_at
Nr2f2	209119_x_at, 209120_at, 209121_x_at, 215073_s_at	209121_x_at
Tgfbi	201506_at	201506_at
Tpm2	204083_s_at, 212654_at	204083_s_at

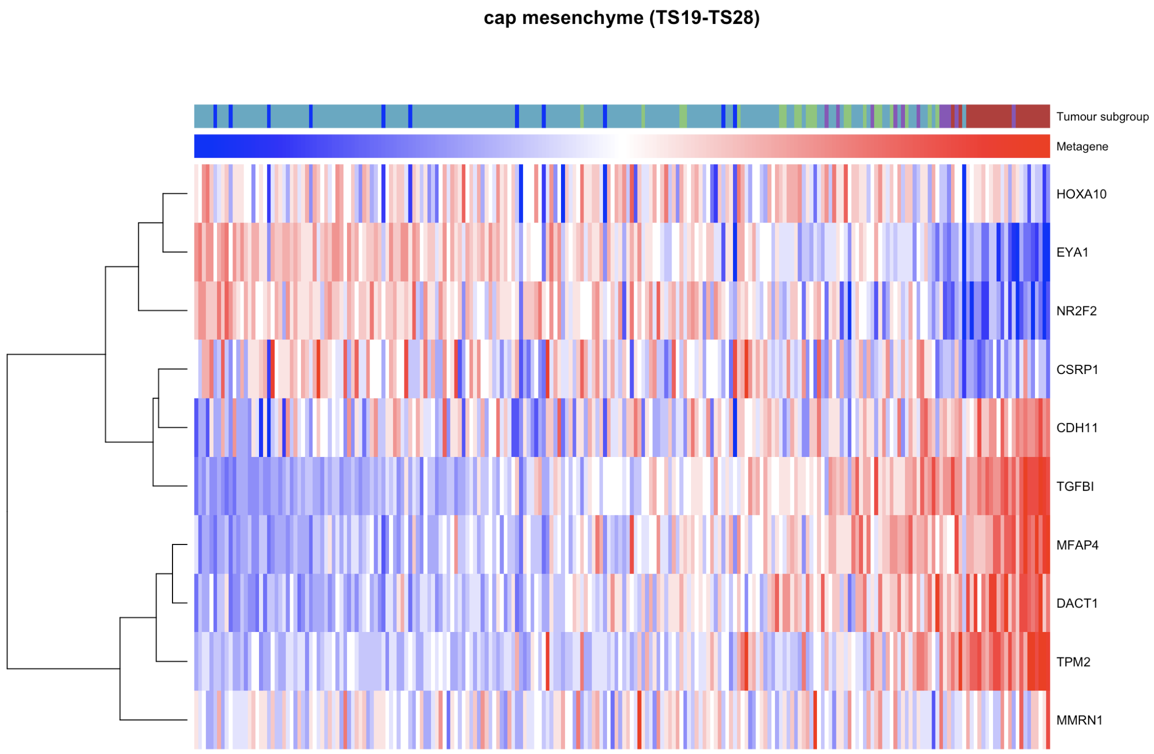

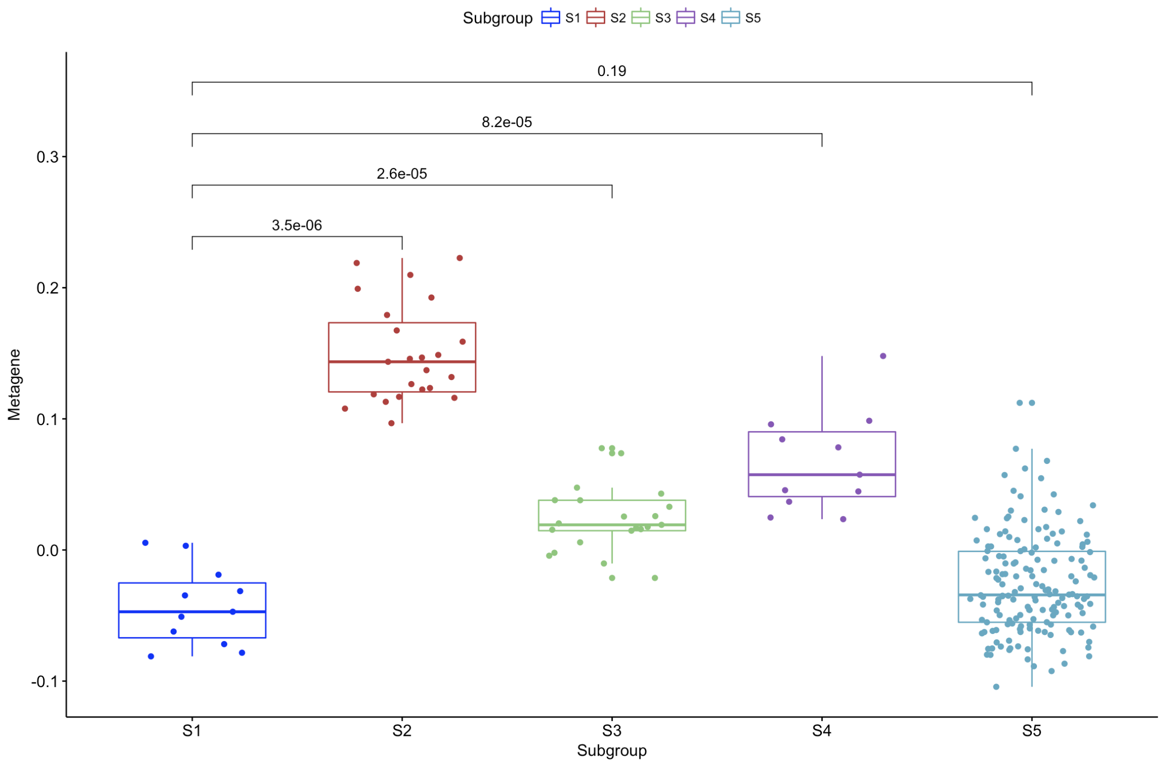

Subgroup
S1
S2
S3
S4
S5
0.19
8.2e-05
2.6e-05
3.5e-06
Metagene
0.3
0.2
0.1
0.0
-0.1
S1
S2
S3
S4
S5
Subgroup

pretubular aggregate (TS20-TS28)

Gene	Probes	Selected
Ccnd1	208711_s_at, 208712_at	208712_at
Cdh4	206866_at, 220227_at	206866_at
Dll1		
Greb1	205862_at, 210562_at, 210855_at	210855_at
Lhx1	206230_at	206230_at
Tmem100	219230_at	219230_at
Wnt4	208606_s_at	208606_s_at
Wt1	206067_s_at, 216953_s_at	206067_s_at

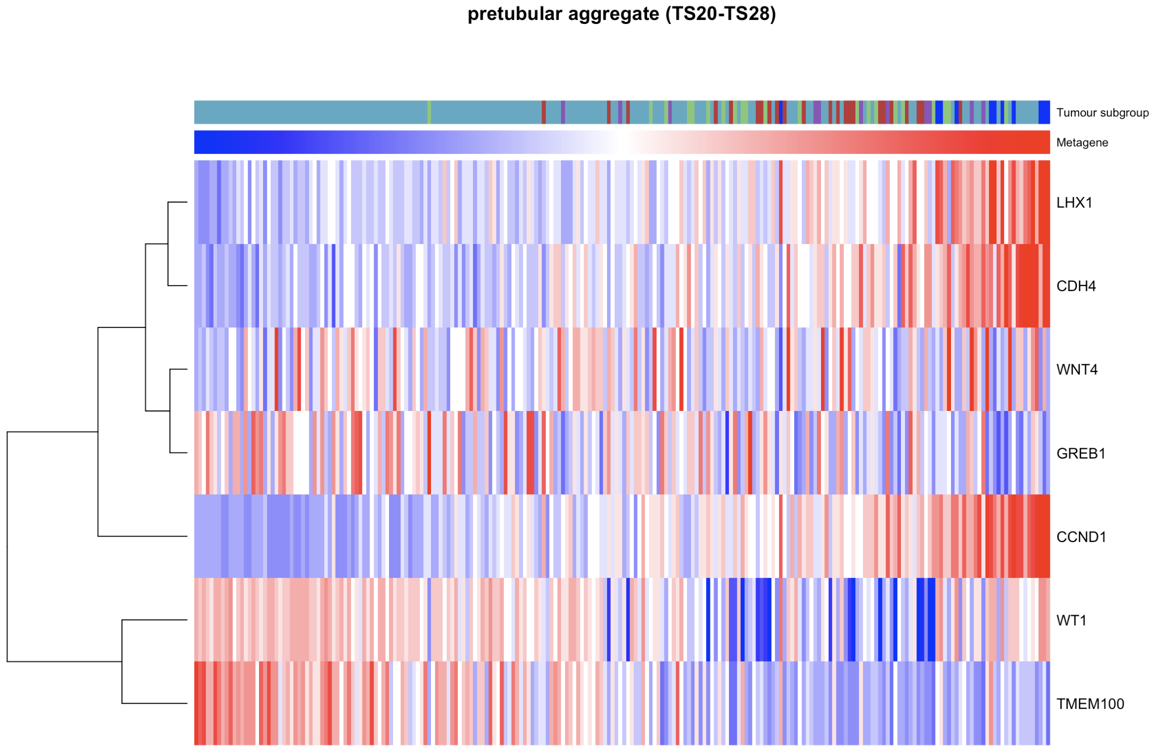

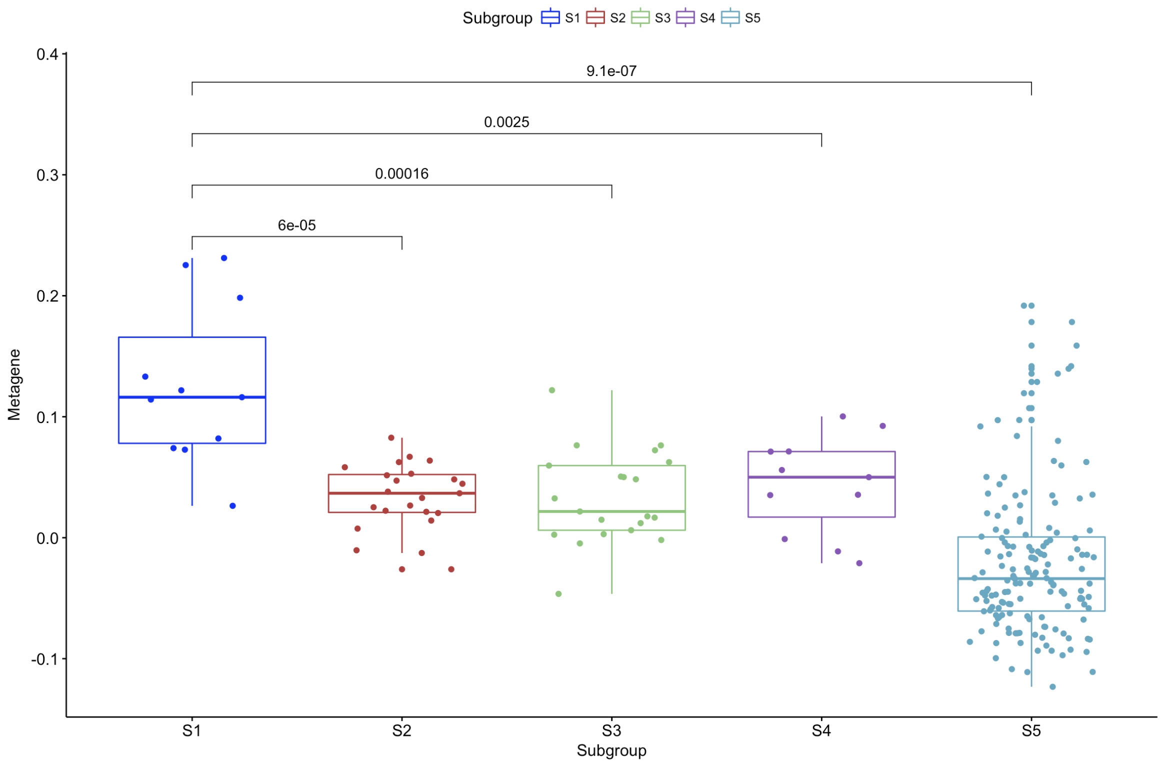

ureteric tip (TS18-TS28)

Gene	Probes	Selected
9230110F11Rik		
Kitl		
Sox9	202935_s_at, 202936_s_at	202936_s_at

NOT ENOUGH GENES FOR METAGENE

stage I / stage II nephron (TS20-TS27)

Gene	Probes	Selected
Bmp2	205289_at, 205290_s_at	205290_s_at
Ccnd1	208711_s_at, 208712_at	208712_at
Cdh4	206866_at, 220227_at	206866_at
Cdh6	205532_s_at, 205533_s_at, 210601_at, 210602_s_at, 214803_at	214803_at
Dkk1	204602_at	204602_at
Dll1		
Greb1	205862_at, 210562_at, 210855_at	210855_at
Jag1	209097_s_at, 209098_s_at, 209099_x_at, 216268_s_at	209099_x_at
Lhx1	206230_at	206230_at
Npy	206001_at	206001_at
Papss2	203058_s_at, 203059_s_at, 203060_s_at	203059_s_at
Pcsk9		
Pou3f3	208563_x_at	208563_x_at
Sox9	202935_s_at, 202936_s_at	202936_s_at
Stc2	203438_at, 203439_s_at	203439_s_at
Tmem100	219230_at	219230_at
Wnt4	208606_s_at	208606_s_at
Wt1	206067_s_at, 216953_s_at	206067_s_at

stage I / stage II nephron (TS20-TS27)

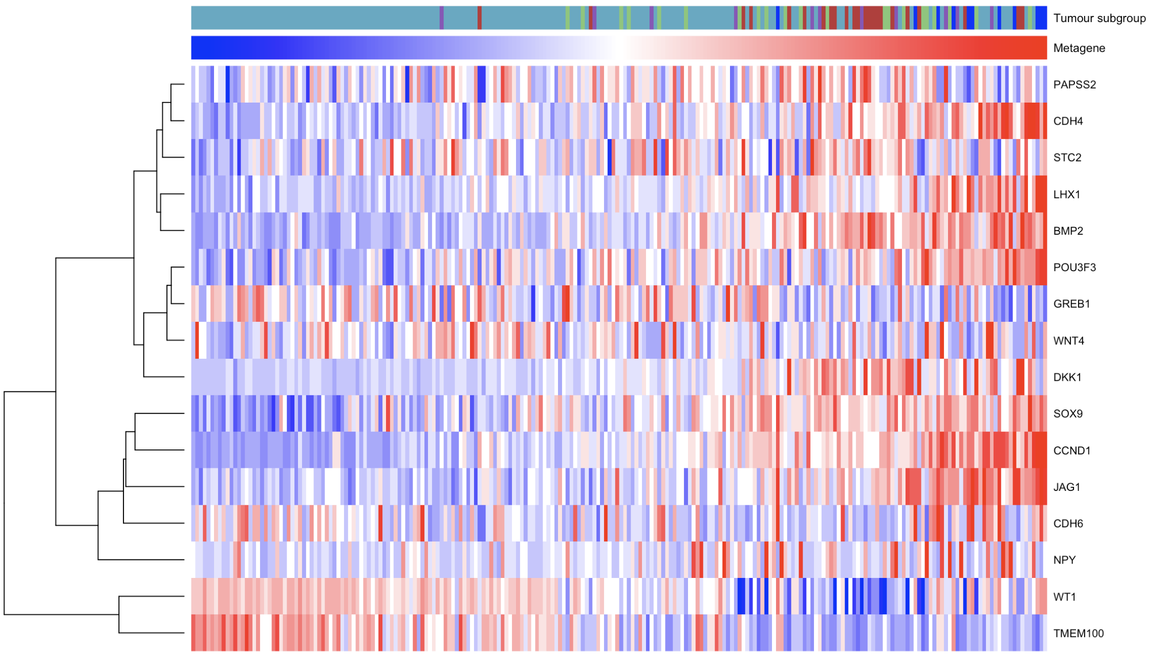

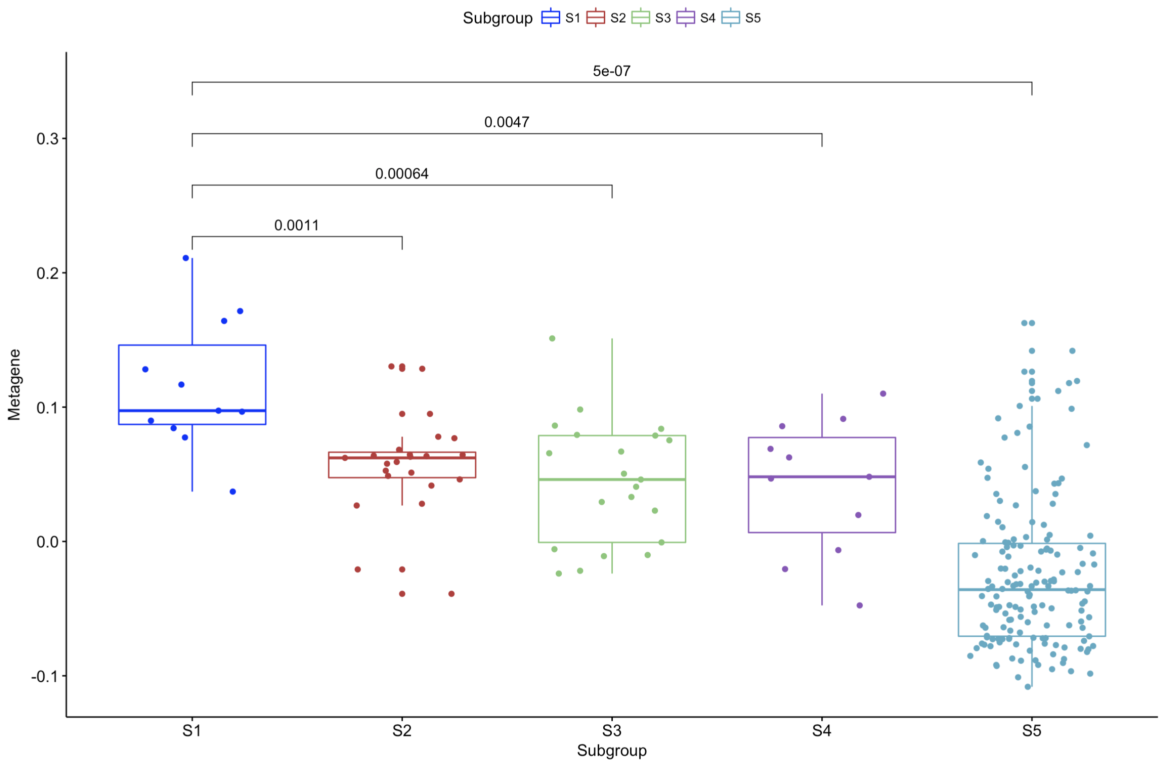

Subgroup S1 S2 S3 S4 S5
5e-07
0.0047
0.00064
0.0011
0.3
0.2
0.1
0.0
-0.1
Metagene
S1
S2
S3
S4
S5
Subgroup

renal vesicle (TS20-TS27)

Gene	Probes	Selected
Bmp2	205289_at, 205290_s_at	205290_s_at
Ccnd1	208711_s_at, 208712_at	208712_at
Cdh4	206866_at, 220227_at	206866_at
Cdh6	205532_s_at, 205533_s_at, 210601_at, 210602_s_at, 214803_at	214803_at
Dkk1	204602_at	204602_at
Dll1		
Greb1	205862_at, 210562_at, 210855_at	210855_at
Jag1	209097_s_at, 209098_s_at, 209099_x_at, 216268_s_at	209099_x_at
Lhx1	206230_at	206230_at
Papss2	203058_s_at, 203059_s_at, 203060_s_at	203059_s_at
Pcsk9		
Pou3f3	208563_x_at	208563_x_at
Sox9	202935_s_at, 202936_s_at	202936_s_at
Tmem100	219230_at	219230_at
Wnt4	208606_s_at	208606_s_at
Wt1	206067_s_at, 216953_s_at	206067_s_at

renal vesicle (TS20-TS27)

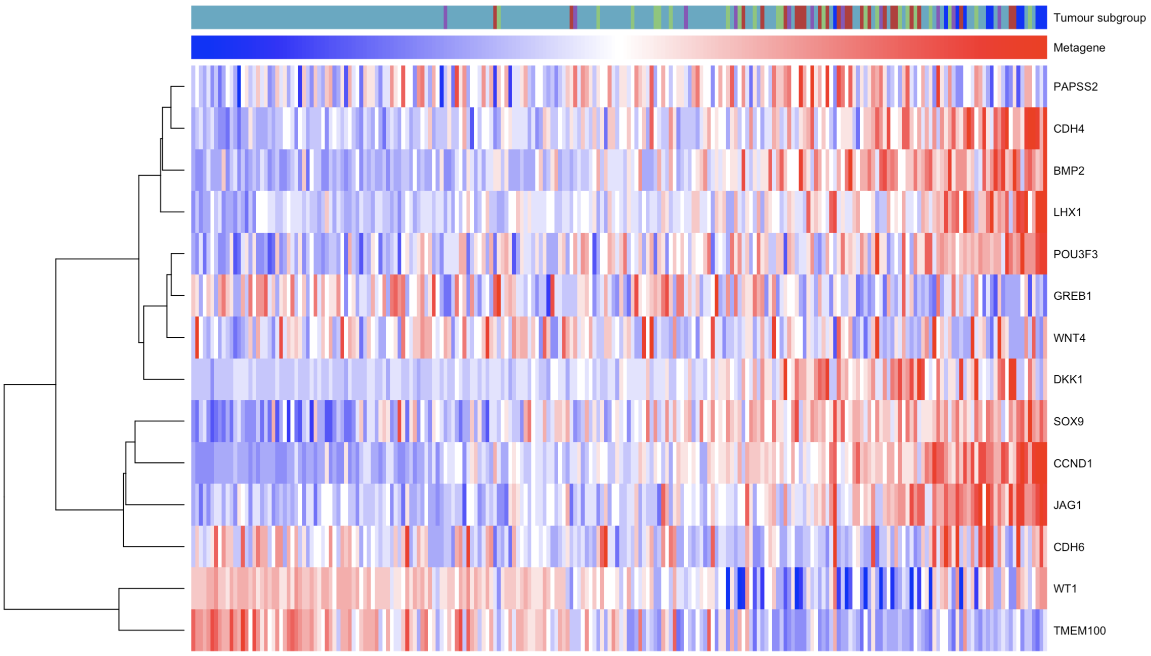

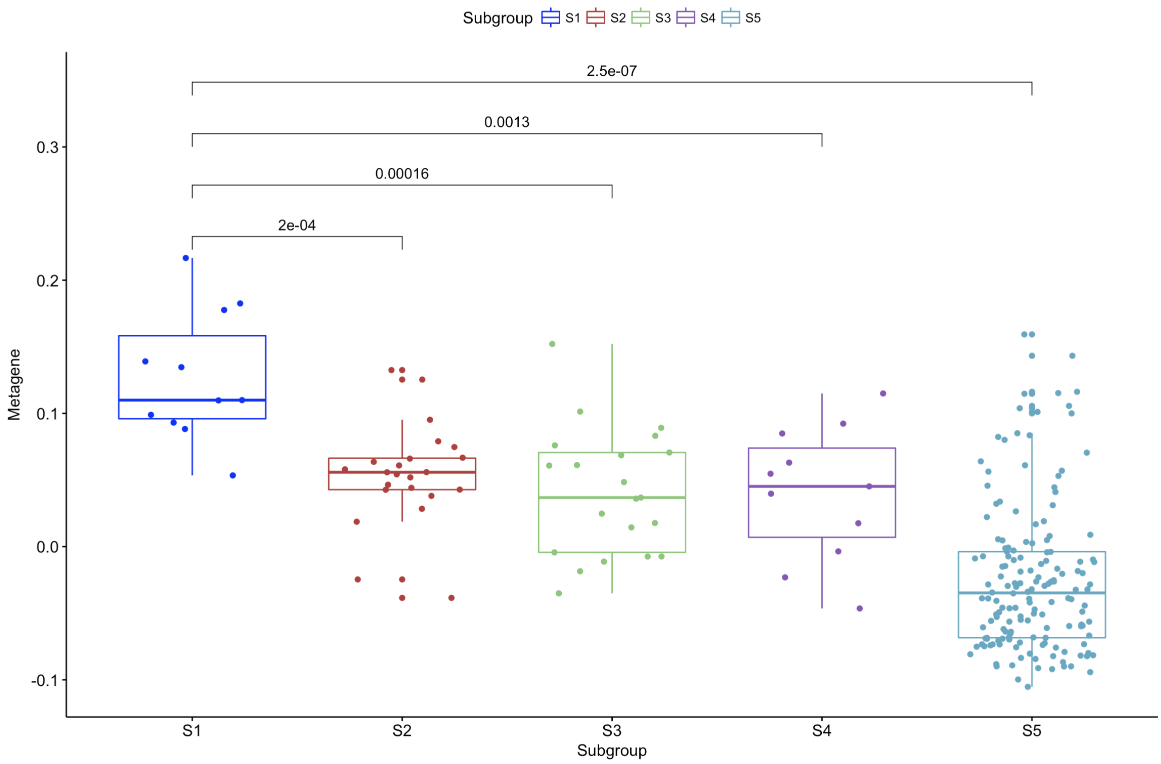

Subgroup
S1
S2
S3
S4
S5
2.5e-07
0.0013
0.00016
2e-04
Metagene
0.3
0.2
0.1
0.0
-0.1
S1
S2
S3
S4
S5
Subgroup

comma-shaped body (TS20-TS27)

Gene	Probes	Selected
Bmp2	205289_at, 205290_s_at	205290_s_at
Ccnd1	208711_s_at, 208712_at	208712_at
Cdh4	206866_at, 220227_at	206866_at
Cdh6	205532_s_at, 205533_s_at, 210601_at, 210602_s_at, 214803_at	214803_at
Dkk1	204602_at	204602_at
Dll1		
Greb1	205862_at, 210562_at, 210855_at	210855_at
Jag1	209097_s_at, 209098_s_at, 209099_x_at, 216268_s_at	209099_x_at
Lhx1	206230_at	206230_at
Papss2	203058_s_at, 203059_s_at, 203060_s_at	203059_s_at
Pcsk9		
Pou3f3	208563_x_at	208563_x_at
Tmem100	219230_at	219230_at
Wnt4	208606_s_at	208606_s_at
Wt1	206067_s_at, 216953_s_at	206067_s_at

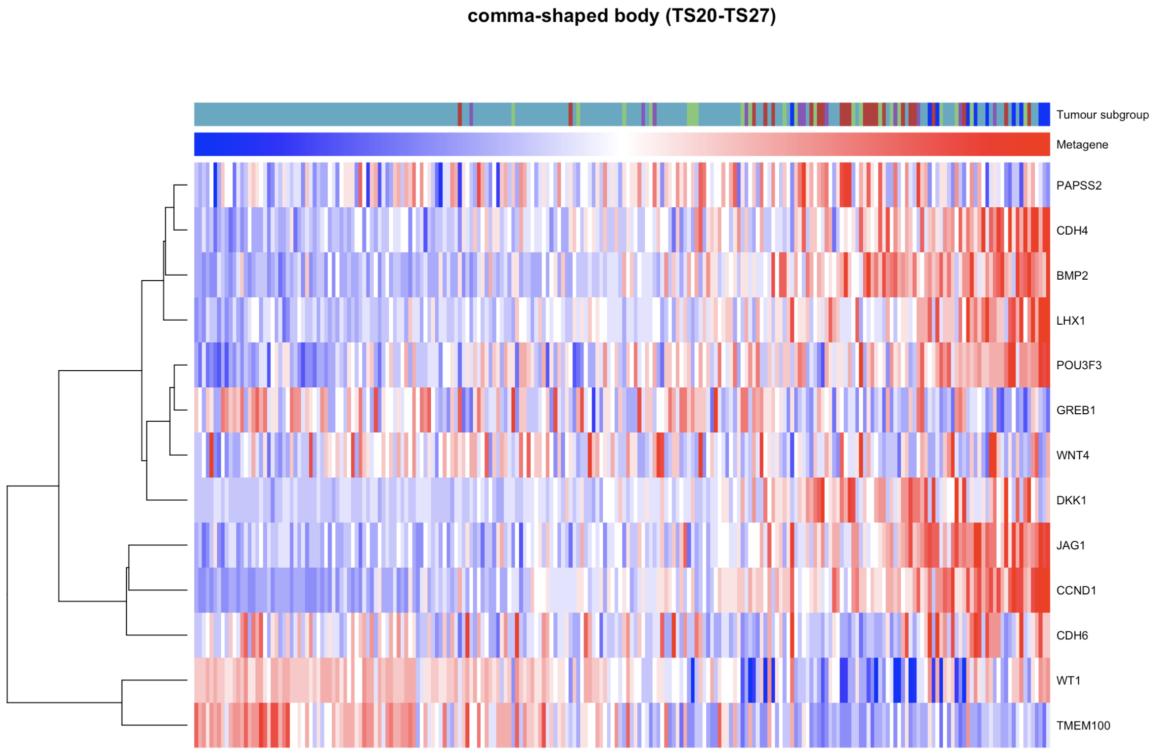

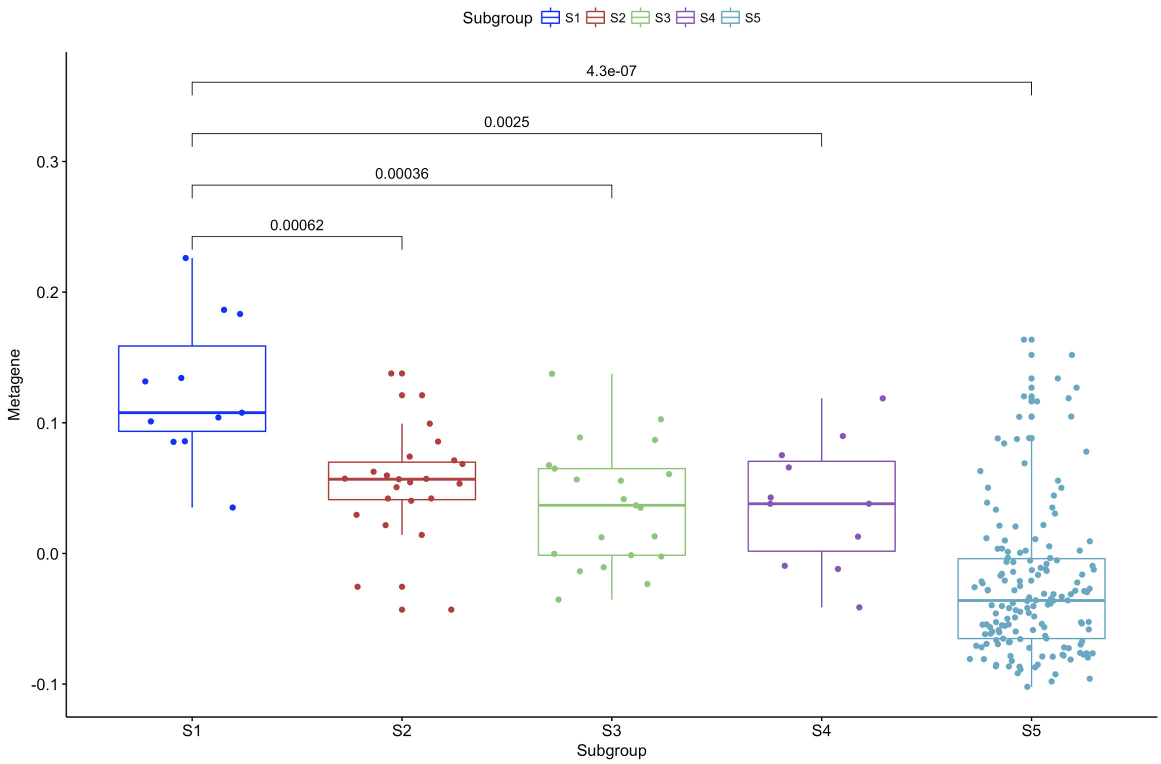
Subgroup S1 S2 S3 S4 S5
4.3e-07
0.0025
0.00036
0.00062
Metagene
0.3
0.2
0.1
0.0
-0.1
S1
S2
S3
S4
S5
Subgroup

s-shaped body (TS21-TS27)

Gene	Probes	Selected
Bmp2	205289_at, 205290_s_at	205290_s_at
Ccnd1	208711_s_at, 208712_at	208712_at
Cdh4	206866_at, 220227_at	206866_at
Cdh6	205532_s_at, 205533_s_at, 210601_at, 210602_s_at, 214803_at	214803_at
Dkk1	204602_at	204602_at
Dll1		
Greb1	205862_at, 210562_at, 210855_at	210855_at
Jag1	209097_s_at, 209098_s_at, 209099_x_at, 216268_s_at	209099_x_at
Lhx1	206230_at	206230_at
Papss2	203058_s_at, 203059_s_at, 203060_s_at	203059_s_at
Pcsk9		
Pou3f3	208563_x_at	208563_x_at
Sox9	202935_s_at, 202936_s_at	202936_s_at
Stc2	203438_at, 203439_s_at	203439_s_at
Tmem100	219230_at	219230_at
Wnt4	208606_s_at	208606_s_at
Wt1	206067_s_at, 216953_s_at	206067_s_at

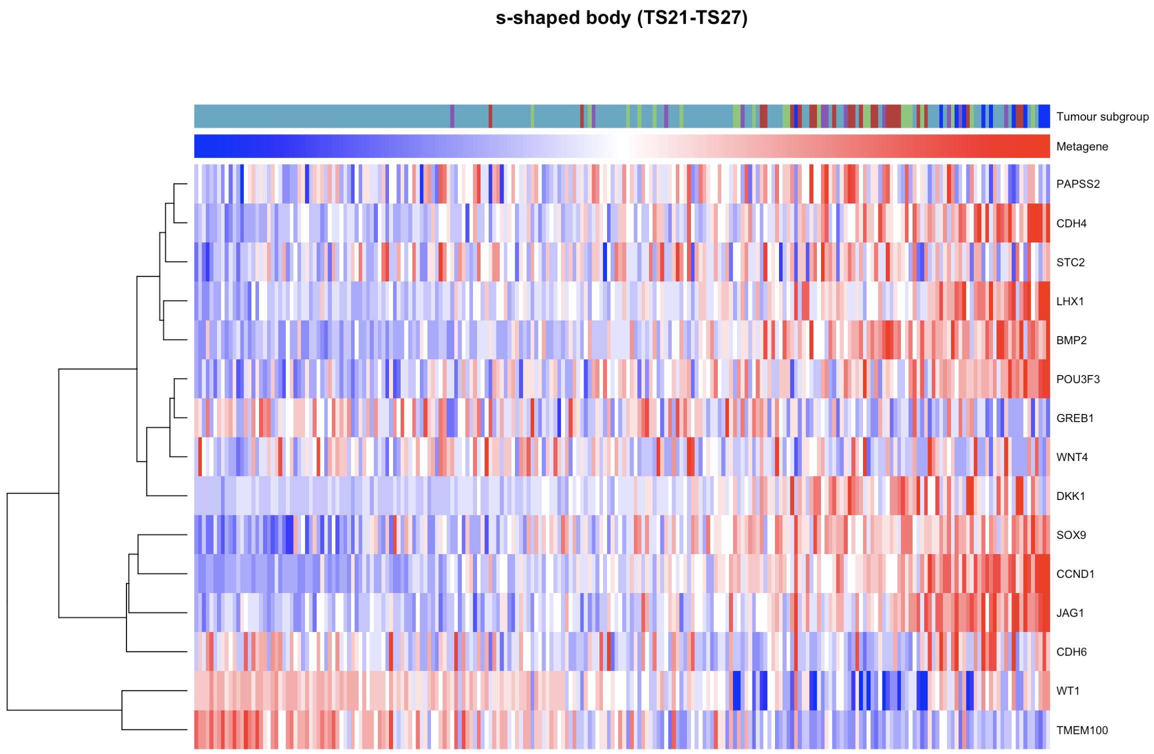

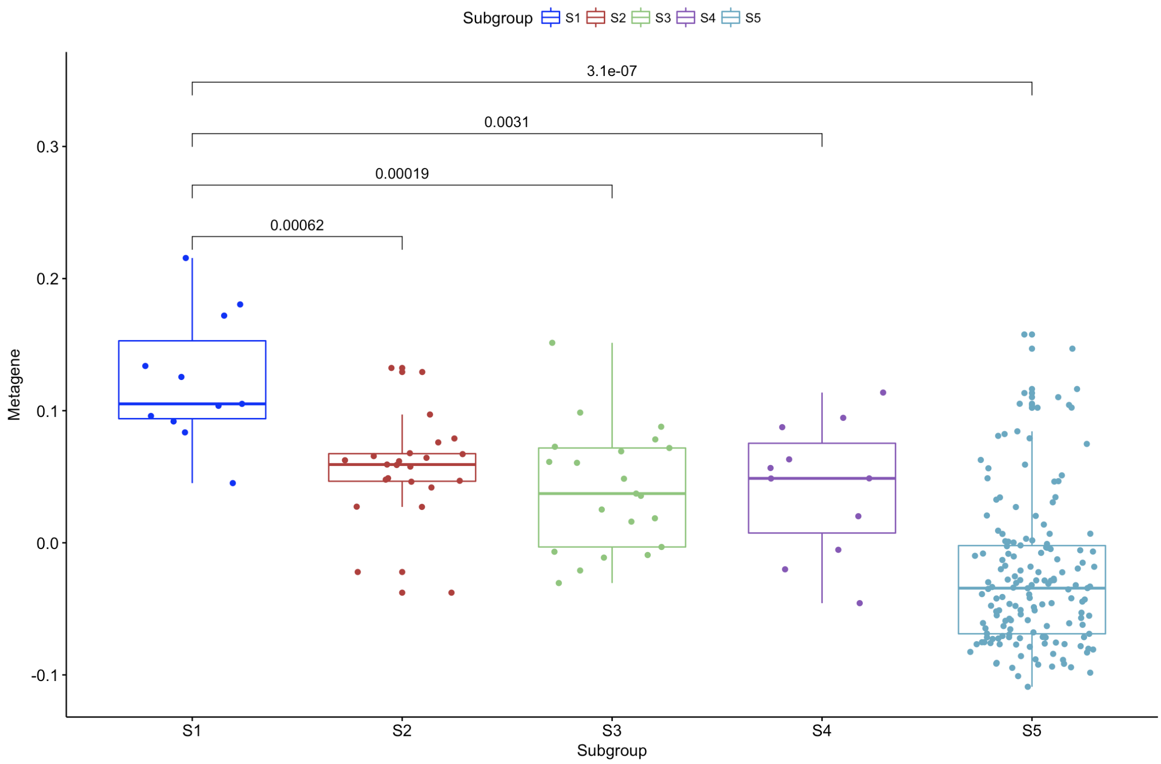

Subgroup S1 S2 S3 S4 S5
3.1e-07
0.0031
0.00019
0.00062
Metagene
0.3
0.2
0.1
0.0
-0.1
S1
S2
S3
S4
S5
Subgroup

stage III / stage IV nephron (TS22-TS28)

Gene	Probes	Selected
0610005C13Rik		
2310046K01Rik		
Aadac	205969_at	205969_at
Acaa1b		
Acsm1	215432_at	215432_at
Aldh1l1	205208_at, 215798_at	205208_at
Ambp	205477_s_at, 214425_at	205477_s_at
Angpt2	205572_at, 211148_s_at	205572_at
Apom	205682_x_at, 214910_s_at	214910_s_at
Bdh2	218285_s_at	218285_s_at
Cgref1	205937_at	205937_at
Cideb	221188_s_at	221188_s_at
Clic6		
Cryl1	220753_s_at	220753_s_at
Dpep1	205983_at	205983_at
Eral1	212087_s_at	212087_s_at
Fbp1	209696_at	209696_at
Fbp2	206844_at	206844_at
Fmo2	211726_s_at	211726_s_at
Gpd1	204997_at, 213706_at	213706_at
Gyk		
Hdc	207067_s_at	207067_s_at
Kcnj1	210402_at, 210403_s_at	210402_at
Mettl7b		
Myo15b	219173_at, 59375_at	59375_at
Napsa		
Pipox	221605_s_at	221605_s_at
Pter	218967_s_at	218967_s_at
Scnn1b	205464_at	205464_at
Serpina10	220626_at	220626_at
Sigirr	218921_at, 52940_at	52940_at
Slc23a1		
Slc27a2	205768_s_at, 205769_at	205769_at
Slc3a1	205799_s_at, 205800_at	205800_at
Slc6a13	207184_at	207184_at
Slc6a20b		
Sord	201562_s_at, 201563_at	201563_at
Stc2	203438_at, 203439_s_at	203439_s_at
Susd2		
Susd3		
Tcn2	204043_at	204043_at
Tesc	218872_at	218872_at
Tmem72		
Ttc36		
Ttr	209660_at	209660_at
Ugt2b37		
Umod	206716_at	206716_at
Unc5cl		

Gene	Probes	Selected
Vdr	204253_s_at, 204254_s_at, 204255_s_at, 213692_s_at	213692_s_at

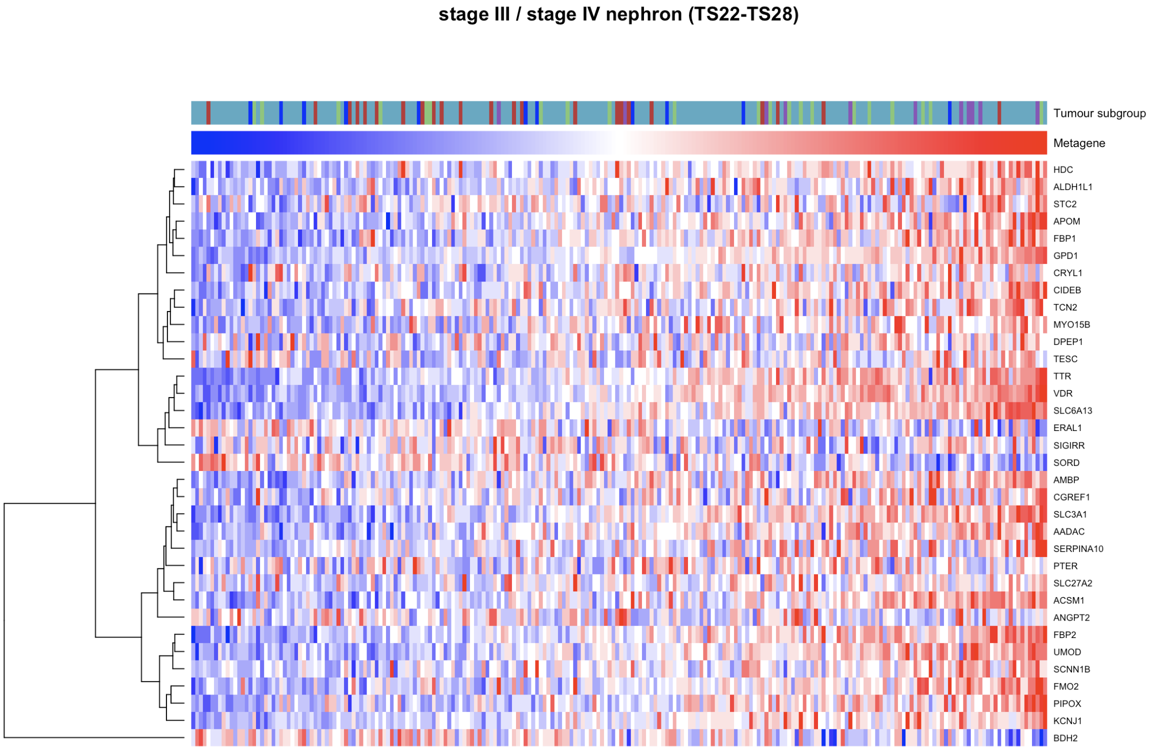

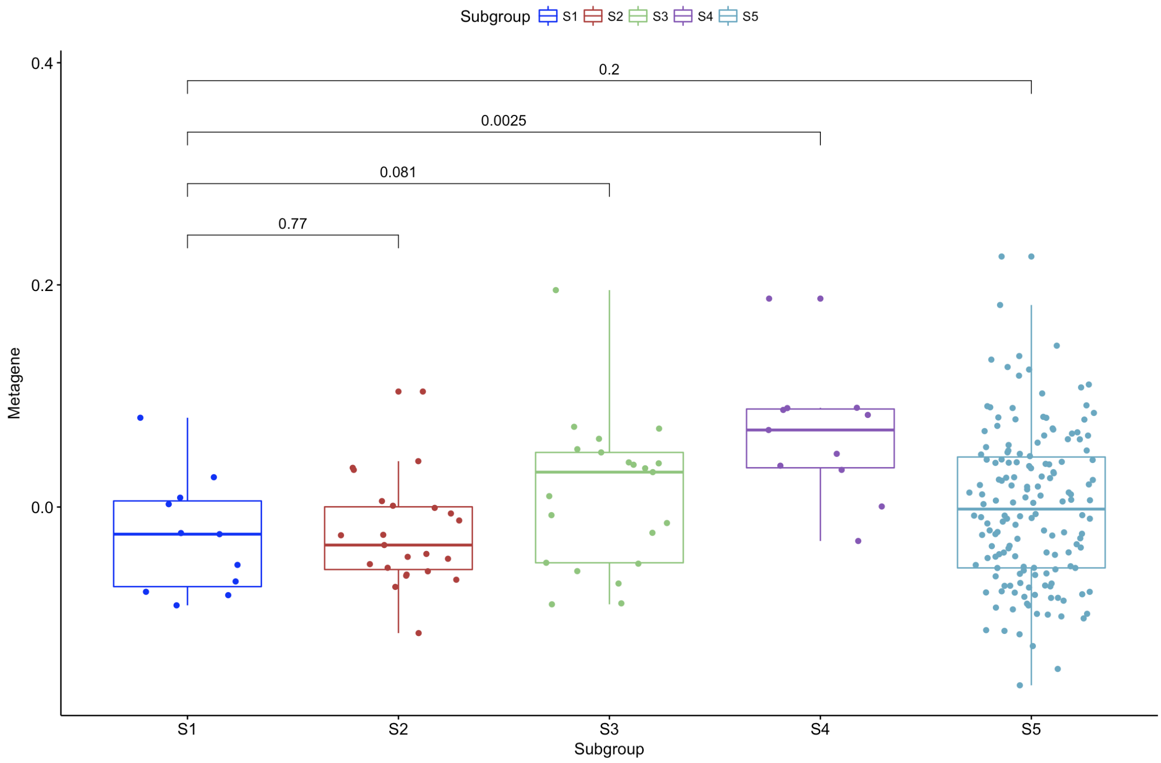

renal proximal tubule (TS25-TS28)

Gene	Probes	Selected
0610005C13Rik		
Acsm1	215432_at	215432_at
Apom	205682_x_at, 214910_s_at	214910_s_at
Bdh2	218285_s_at	218285_s_at
Cgref1	205937_at	205937_at
Cryl1	220753_s_at	220753_s_at
Fbp1	209696_at	209696_at
Fmo2	211726_s_at	211726_s_at
Gyk		
Mettl7b		
Pter	218967_s_at	218967_s_at
Slc27a2	205768_s_at, 205769_at	205769_at
Slc3a1	205799_s_at, 205800_at	205800_at
Slc6a20b		
Sord	201562_s_at, 201563_at	201563_at
Tcn2	204043_at	204043_at
Ugt2b37		
Umod	206716_at	206716_at

renal proximal tubule (TS25-TS28)

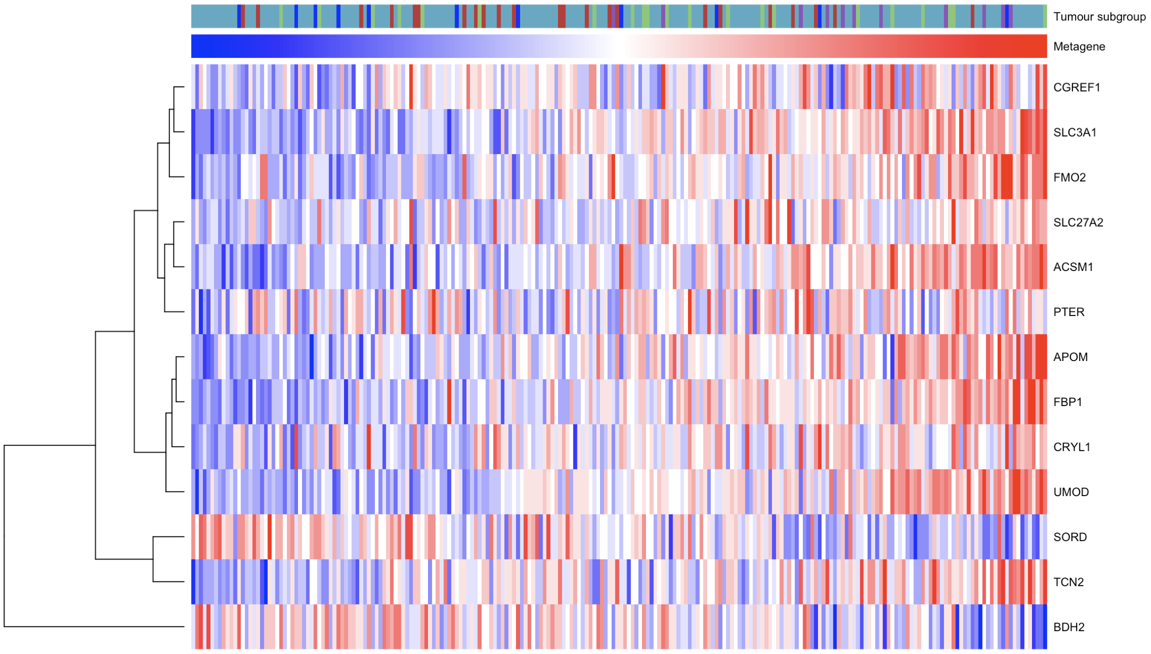

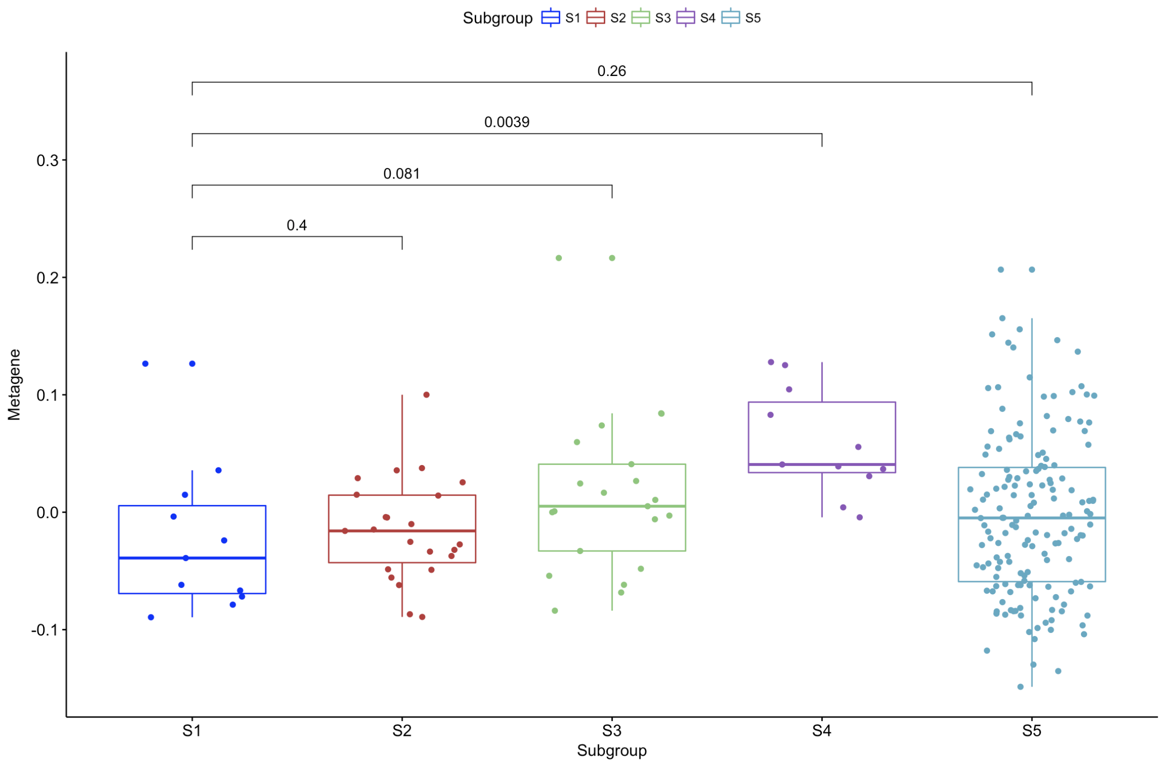

Subgroup S1 S2 S3 S4 S5
0.26
0.0039
0.081
0.4
0.3
0.2
0.1
0.0
-0.1
Metagene
S1
S2
S3
S4
S5
Subgroup

loop of Henle (TS25-TS28)

Gene	Probes	Selected
Umod	206716_at	206716_at

NOT ENOUGH GENES FOR METAGENE

renal distal tubule (TS25-TS28)

Gene	Probes	Selected
Umod	206716_at	206716_at

NOT ENOUGH GENES FOR METAGENE

renal corpuscle (TS21-TS28)

Gene	Probes	Selected
Angpt2	205572_at, 211148_s_at	205572_at
Dpep1	205983_at	205983_at
Eral1	212087_s_at	212087_s_at
Gpd1	204997_at, 213706_at	213706_at
Stc2	203438_at, 203439_s_at	203439_s_at
Susd2		

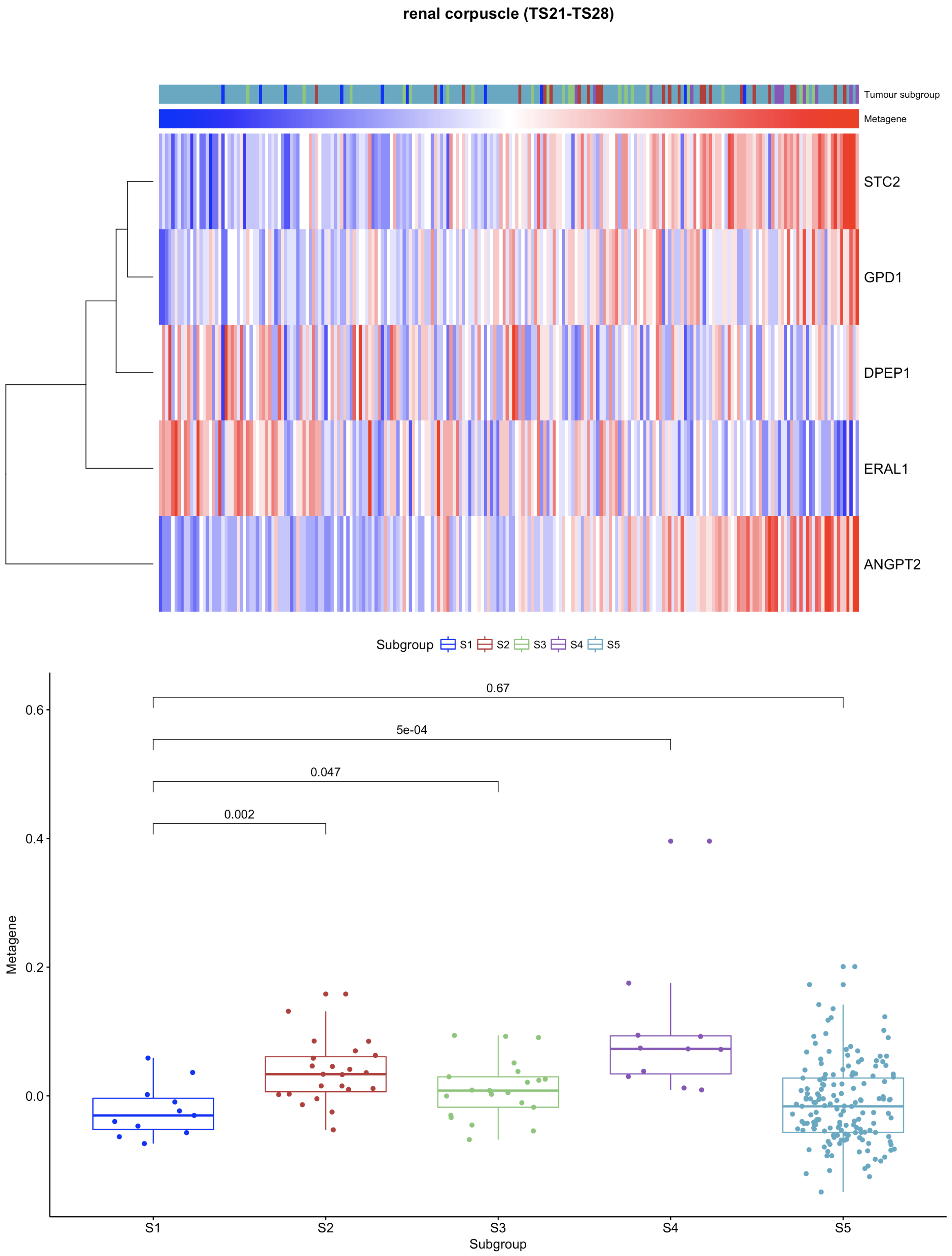

capillary loop stage nephron (stage III) (TS22-TS28)

Gene	Probes	Selected
0610005C13Rik		
2310046K01Rik		
Aadac	205969_at	205969_at
Acaa1b		
Acsm1	215432_at	215432_at
Aldh1l1	205208_at, 215798_at	205208_at
Ambp	205477_s_at, 214425_at	205477_s_at
Apom	205682_x_at, 214910_s_at	214910_s_at
Bdh2	218285_s_at	218285_s_at
Cgref1	205937_at	205937_at
Cideb	221188_s_at	221188_s_at
Clic6		
Cryl1	220753_s_at	220753_s_at
Dpep1	205983_at	205983_at
Eral1	212087_s_at	212087_s_at
Fbp1	209696_at	209696_at
Fbp2	206844_at	206844_at
Fmo2	211726_s_at	211726_s_at
Gpd1	204997_at, 213706_at	213706_at
Gyk		
Hdc	207067_s_at	207067_s_at
Kcnj1	210402_at, 210403_s_at	210402_at
Mettl7b		
Myo15b	219173_at, 59375_at	59375_at
Napsa		
Pipox	221605_s_at	221605_s_at
Pter	218967_s_at	218967_s_at
Scnn1b	205464_at	205464_at
Serpina10	220626_at	220626_at
Sigirr	218921_at, 52940_at	52940_at
Slc23a1		
Slc27a2	205768_s_at, 205769_at	205769_at
Slc3a1	205799_s_at, 205800_at	205800_at
Slc6a13	207184_at	207184_at
Slc6a20b		
Sord	201562_s_at, 201563_at	201563_at
Stc2	203438_at, 203439_s_at	203439_s_at
Susd2		
Susd3		
Tcn2	204043_at	204043_at
Tesc	218872_at	218872_at
Tmem72		
Ttc36		
Ttr	209660_at	209660_at
Ugt2b37		
Umod	206716_at	206716_at
Unc5cl		
Vdr	204253_s_at, 204254_s_at, 204255_s_at, 213692_s_at	213692_s_at

capillary loop stage nephron (stage III) (TS22-TS28)

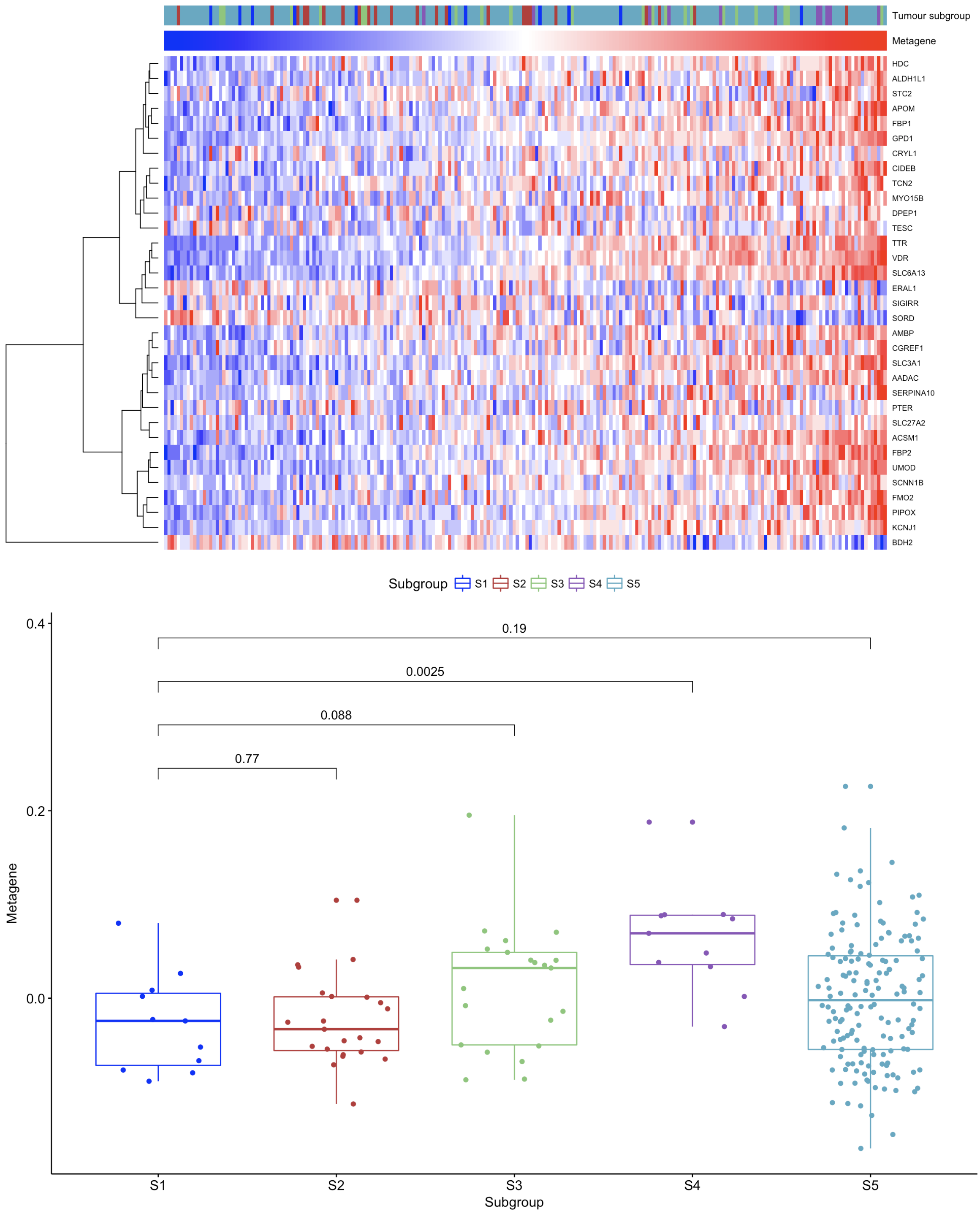

maturing nephron (stage IV) (TS22-TS28)

Gene	Probes	Selected
0610005C13Rik		
2310046K01Rik		
Aadac	205969_at	205969_at
Acaa1b		
Acsm1	215432_at	215432_at
Aldh1l1	205208_at, 215798_at	205208_at
Ambp	205477_s_at, 214425_at	205477_s_at
Angpt2	205572_at, 211148_s_at	205572_at
Apom	205682_x_at, 214910_s_at	214910_s_at
Bdh2	218285_s_at	218285_s_at
Cgref1	205937_at	205937_at
Cideb	221188_s_at	221188_s_at
Clic6		
Cryl1	220753_s_at	220753_s_at
Dpep1	205983_at	205983_at
Eral1	212087_s_at	212087_s_at
Fbp1	209696_at	209696_at
Fbp2	206844_at	206844_at
Fmo2	211726_s_at	211726_s_at
Gpd1	204997_at, 213706_at	213706_at
Gyk		
Hdc	207067_s_at	207067_s_at
Kcnj1	210402_at, 210403_s_at	210402_at
Mettl7b		
Myo15b	219173_at, 59375_at	59375_at
Napsa		
Pipox	221605_s_at	221605_s_at
Pter	218967_s_at	218967_s_at
Scnn1b	205464_at	205464_at
Serpina10	220626_at	220626_at
Sigirr	218921_at, 52940_at	52940_at
Slc23a1		
Slc27a2	205768_s_at, 205769_at	205769_at
Slc3a1	205799_s_at, 205800_at	205800_at
Slc6a13	207184_at	207184_at
Slc6a20b		
Sord	201562_s_at, 201563_at	201563_at
Stc2	203438_at, 203439_s_at	203439_s_at
Susd2		
Susd3		
Tcn2	204043_at	204043_at
Tesc	218872_at	218872_at
Tmem72		
Ttc36		
Ttr	209660_at	209660_at
Ugt2b37		
Umod	206716_at	206716_at
Unc5cl		

Gene	Probes	Selected
Vdr	204253_s_at, 204254_s_at, 204255_s_at, 213692_s_at	213692_s_at

maturing nephron (stage IV) (TS22-TS28)

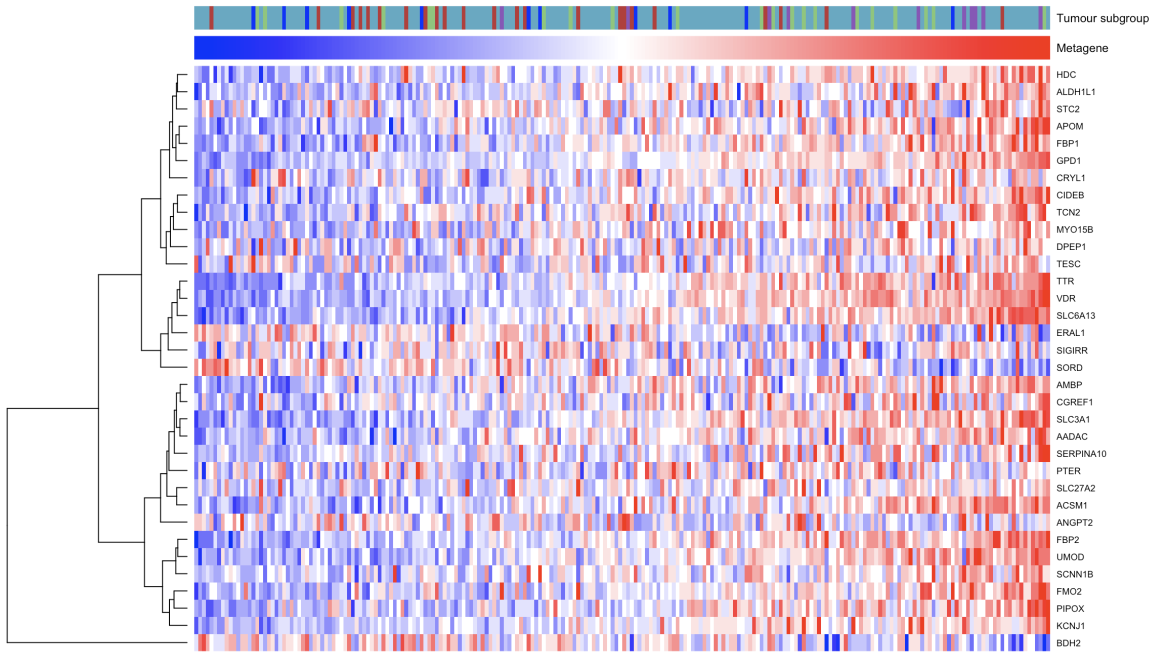

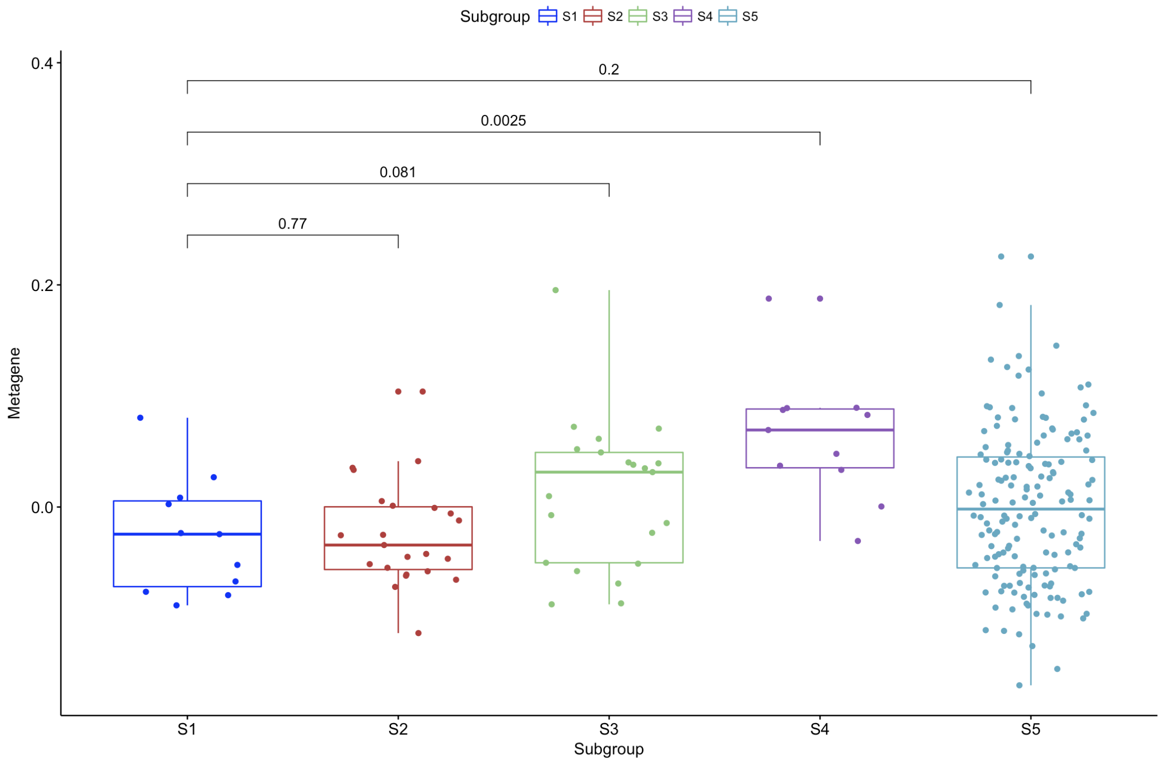

collecting duct (TS21-TS28)

Gene	Probes	Selected
9230110F11Rik		
Capg	201850_at	201850_at
Kitl		
Ppp1r3c	204284_at	204284_at
Scnn1b	205464_at	205464_at
Sox9	202935_s_at, 202936_s_at	202936_s_at
Tesc	218872_at	218872_at
Upk1b	210064_s_at, 210065_s_at	210064_s_at
Vldlr	209822_s_at	209822_s_at

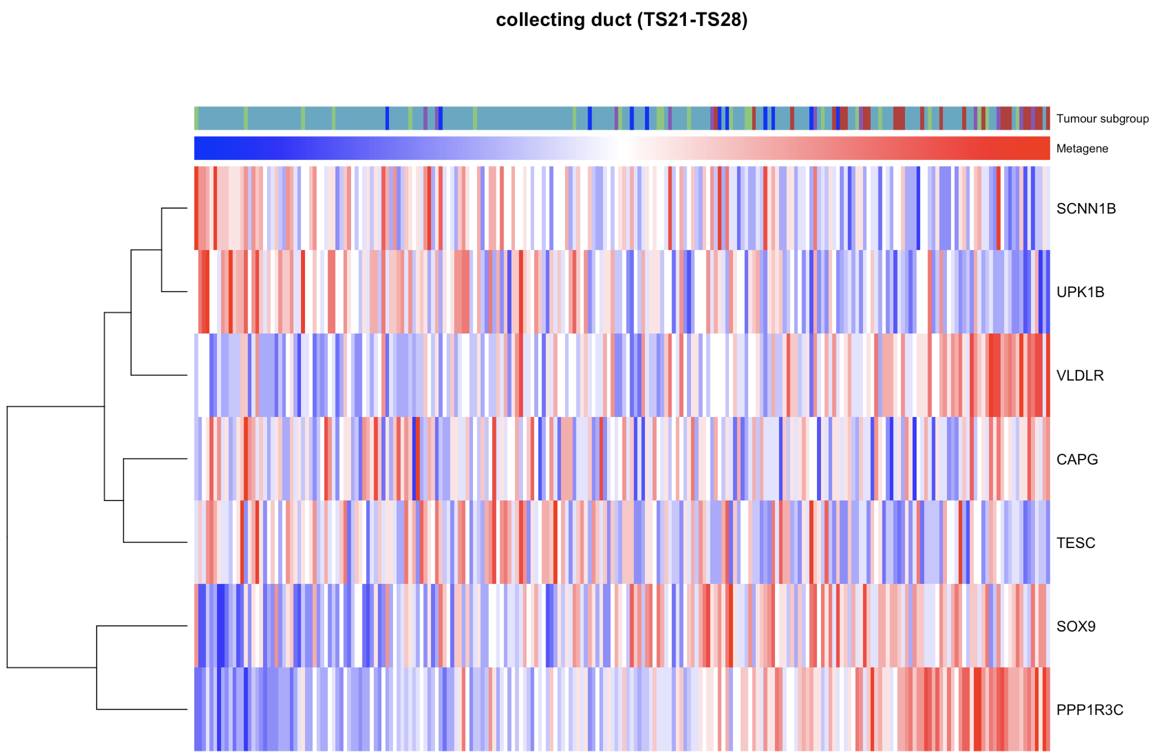

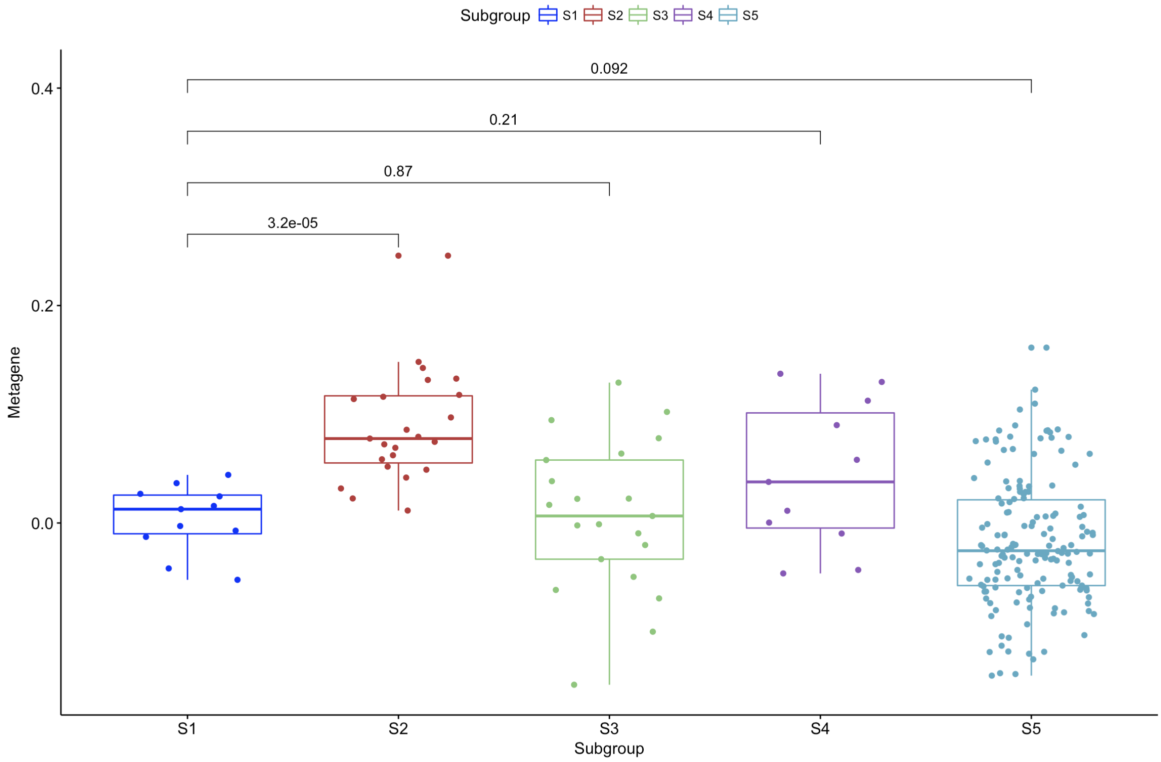

medullary collecting duct (TS22-TS28)

Gene	Probes	Selected
Ppp1r3c	204284_at	204284_at
Upk1b	210064_s_at, 210065_s_at	210064_s_at

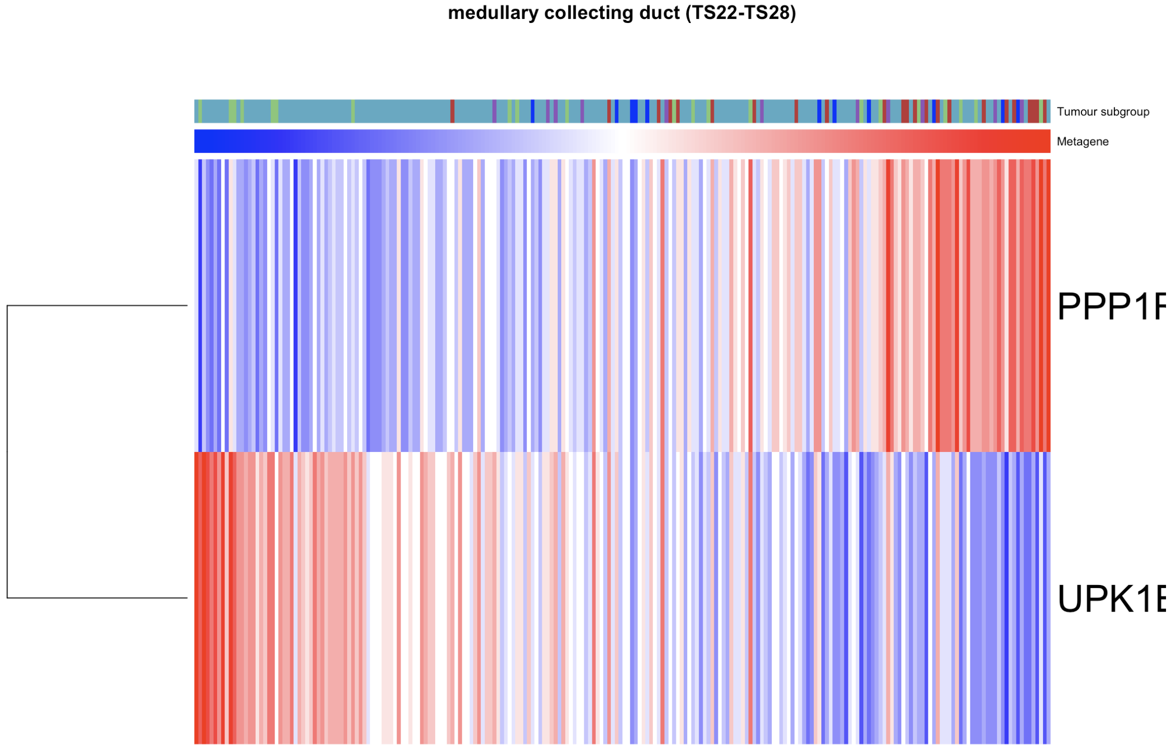

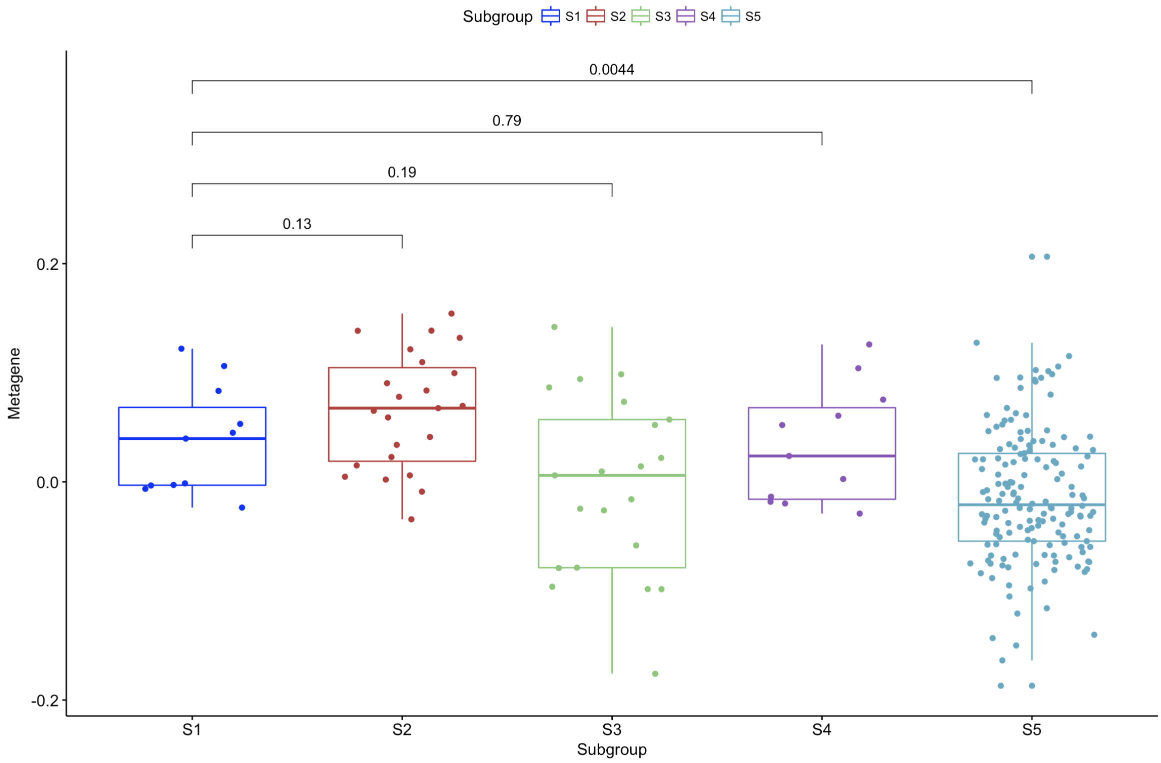

renal interstitium (TS20-TS28)

Gene	Probes	Selected
Aff3	205734_s_at, 205735_s_at	205735_s_at
Cdh11	207172_s_at, 207173_x_at	207173_x_at
Col23a1		
Col3a1	201852_x_at, 211161_s_at, 215076_s_at, 215077_at	215076_s_at
Col9a1	222008_at	222008_at
Csrp1	200621_at	200621_at
Dact1	219179_at	219179_at
Dnm3os		
Dzip1	204556_s_at, 204557_s_at	204556_s_at
Emilin1	204163_at	204163_at
Gria4	208464_at	208464_at
Gulp1	204235_s_at, 204237_at, 215913_s_at, 215915_at	215913_s_at
Hoxa10	213147_at, 213150_at	213150_at
Igf1	209540_at, 209541_at, 209542_x_at, 211577_s_at	211577_s_at
Lgals1	201105_at	201105_at
Lix1l		
Mfap4	212713_at	212713_at
Mll5		
Mmrn1	205612_at	205612_at
Ms4a6b		
Myl9	201058_s_at	201058_s_at
Nr2f2	209119_x_at, 209120_at, 209121_x_at, 215073_s_at	209121_x_at
Olfml3	218162_at	218162_at
Pdgfra	203131_at, 211533_at, 215305_at	203131_at
Pid1	219093_at	219093_at
Prickle2		
S1pr3		
Synpo2		
Tgfb1i1	209651_at	209651_at
Tgfbi	201506_at	201506_at
Tgm5	207911_s_at	207911_s_at
Tpm2	204083_s_at, 212654_at	204083_s_at
Tshz3		

renal interstitium (TS20-TS28)

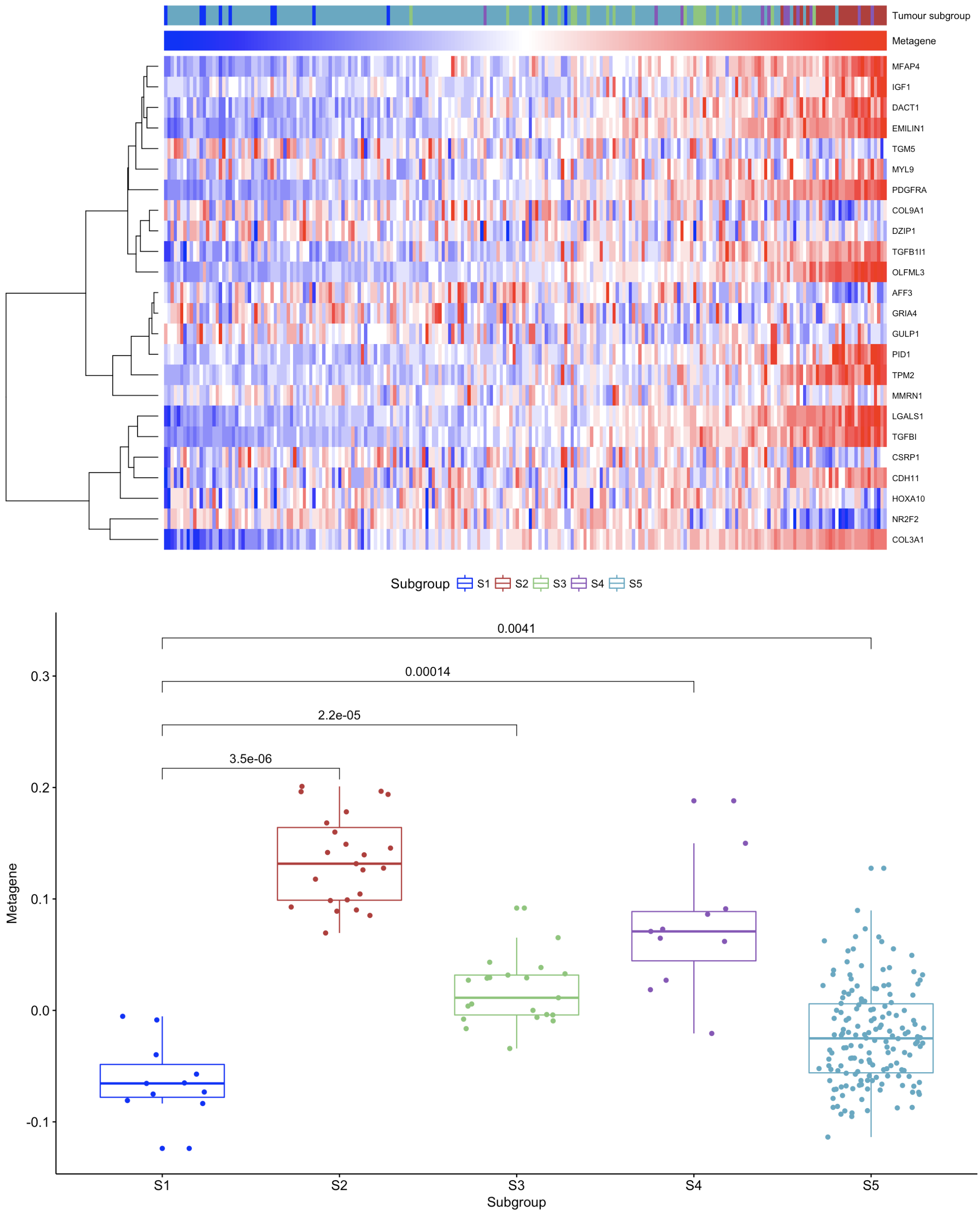

immature loop of Henle (TS22-TS24)

Gene	Probes	Selected
0610005C13Rik		
2310046K01Rik		
Aldh1l1	205208_at, 215798_at	205208_at
Ambp	205477_s_at, 214425_at	205477_s_at
Clic6		
Fbp2	206844_at	206844_at
Gpd1	204997_at, 213706_at	213706_at
Gyk		
Kcnj1	210402_at, 210403_s_at	210402_at
Napsa		
Pter	218967_s_at	218967_s_at
Serpina10	220626_at	220626_at
Sigirr	218921_at, 52940_at	52940_at
Slc23a1		
Susd2		
Tmem72		
Ttr	209660_at	209660_at

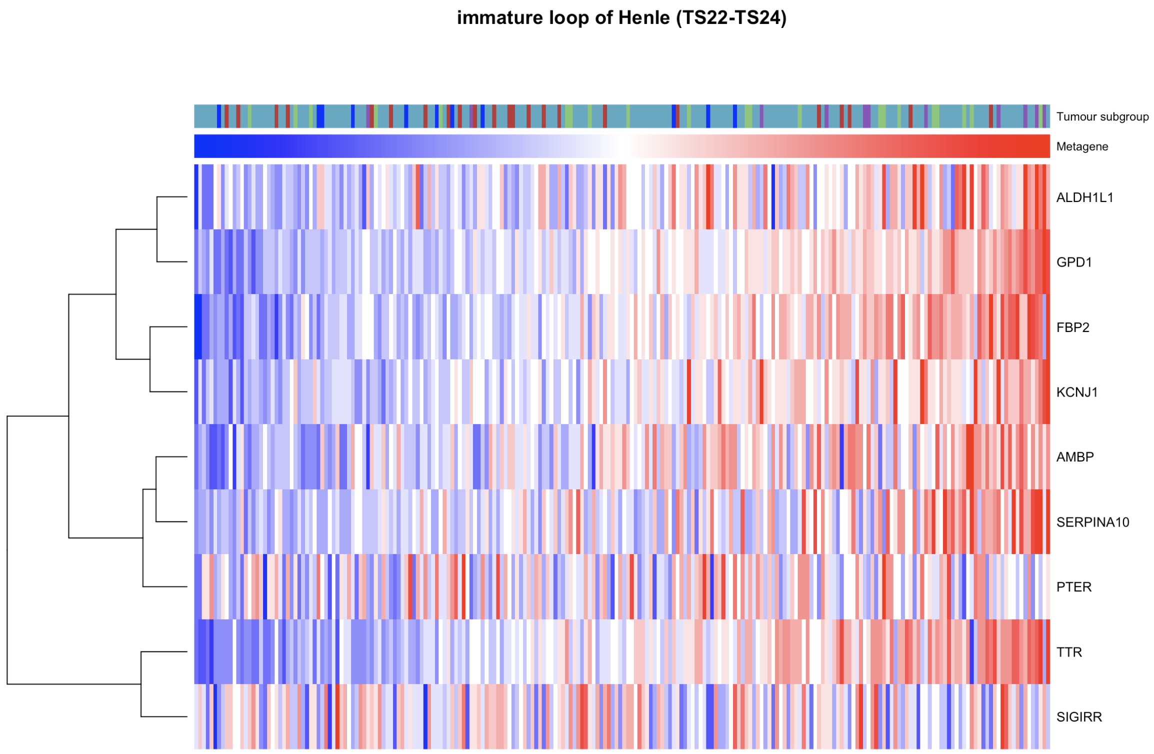

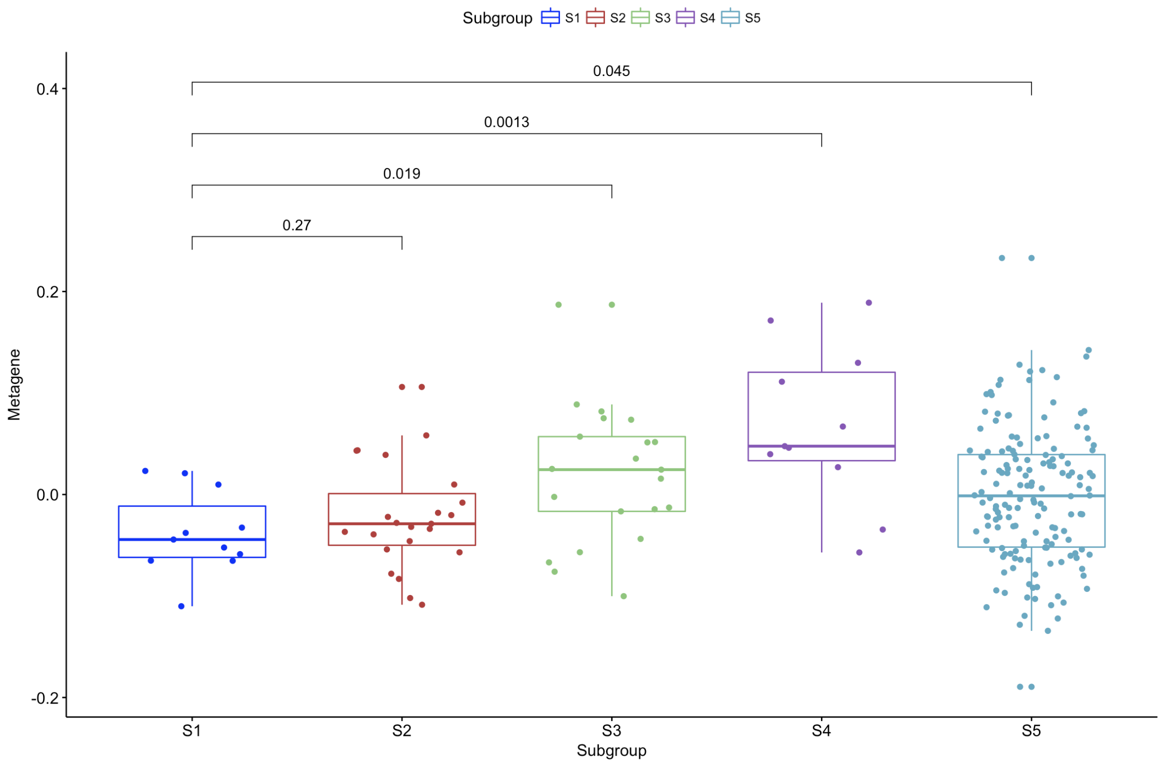
Subgroup S1 S2 S3 S4 S5
0.045
0.0013
0.019
0.27
Metagene
0.4
0.2
0.0
-0.2
S1
S2
S3
S4
S5
Subgroup

early distal tubule (TS21-TS27)

Gene	Probes	Selected
Aldh1l1	205208_at, 215798_at	205208_at
Gyk		
Kcnj1	210402_at, 210403_s_at	210402_at
Scnn1b	205464_at	205464_at
Sigirr	218921_at, 52940_at	52940_at
Tmem72		

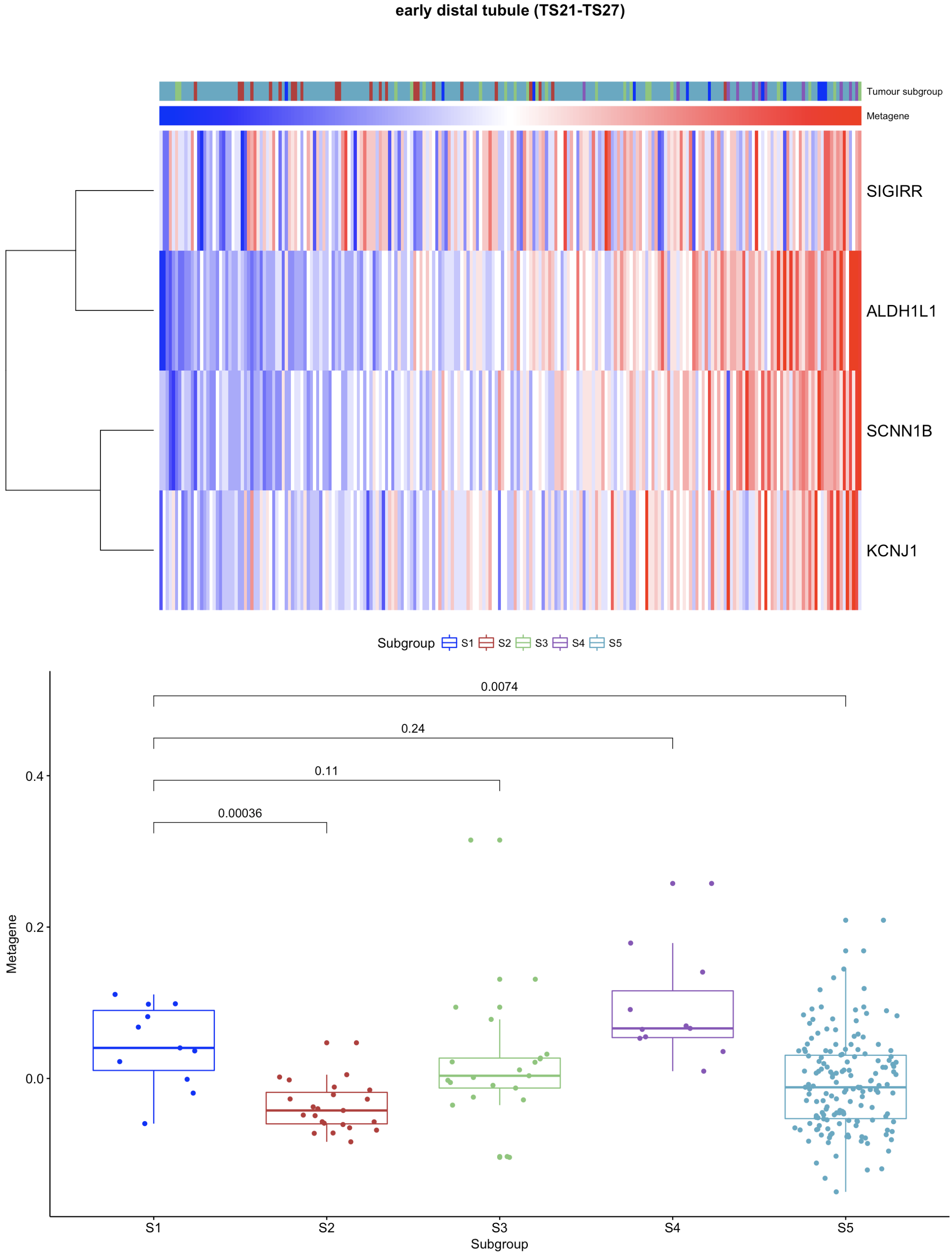

Supplement: S7 Fig — (PDF) [file pgen.1007399.s011.pdf]
